# Heterosynaptic Plasticity and the Experience-Dependent Refinement of Developing Neuronal Circuits

**DOI:** 10.3389/fncir.2021.803401

**Published:** 2021-12-07

**Authors:** Kyle R. Jenks, Katya Tsimring, Jacque Pak Kan Ip, Jose C. Zepeda, Mriganka Sur

**Affiliations:** ^1^Picower Institute for Learning and Memory, Department of Brain and Cognitive Sciences, Massachusetts Institute of Technology, Cambridge, MA, United States; ^2^School of Biomedical Sciences, The Chinese University of Hong Kong, Hong Kong SAR, China

**Keywords:** synapses, development, heterosynaptic plasticity, critical period, ocular dominance, visual cortex

## Abstract

Neurons remodel the structure and strength of their synapses during critical periods of development in order to optimize both perception and cognition. Many of these developmental synaptic changes are thought to occur through synapse-specific homosynaptic forms of experience-dependent plasticity. However, homosynaptic plasticity can also induce or contribute to the plasticity of neighboring synapses through heterosynaptic interactions. Decades of research *in vitro* have uncovered many of the molecular mechanisms of heterosynaptic plasticity that mediate local compensation for homosynaptic plasticity, facilitation of further bouts of plasticity in nearby synapses, and cooperative induction of plasticity by neighboring synapses acting in concert. These discoveries greatly benefited from new tools and technologies that permitted single synapse imaging and manipulation of structure, function, and protein dynamics in living neurons. With the recent advent and application of similar tools for *in vivo* research, it is now feasible to explore how heterosynaptic plasticity contribute to critical periods and the development of neuronal circuits. In this review, we will first define the forms heterosynaptic plasticity can take and describe our current understanding of their molecular mechanisms. Then, we will outline how heterosynaptic plasticity may lead to meaningful refinement of neuronal responses and observations that suggest such mechanisms are indeed at work *in vivo*. Finally, we will use a well-studied model of cortical plasticity—ocular dominance plasticity during a critical period of visual cortex development—to highlight the molecular overlap between heterosynaptic and developmental forms of plasticity, and suggest potential avenues of future research.

## Introduction

Experience refines the connectivity of neuronal circuits during critical periods in development when the plasticity of the synaptic connections between neurons peaks. Identifying what forms of plasticity are evoked by experience and how these changes, in turn, lead to the development of perception and cognition is challenging in the context of an intact and ever-changing neuronal circuit. However, disambiguating the contribution of distinct forms of plasticity is crucial both to understand the normal development of the brain and how disruption of plasticity during development leads to neurodevelopmental disorders (Dakin and Frith, 2005; Leblanc and Fagiolini, 2011; Meredith, 2015). Decades of research into the molecular mechanisms of synaptic plasticity *in vitro* have proven to be the key to addressing these questions *in vivo*, by allowing us to ask where, when, and under what conditions the molecular mechanisms underlying specific forms of plasticity are required.

Unique classes of plasticity alter synaptic strength bidirectionally with varying degrees of specificity and at varying spatial and temporal scales. Homosynaptic (also known as Hebbian) forms of plasticity were the first to be identified, and rapidly alter the synapses activated by the plasticity-inducing stimulus itself. Homosynaptic long-term potentiation (LTP) and depression (LTD) thereby mediate input-specific changes and are widely accepted to be the synaptic correlates of learning and memory (Bliss and Collingridge, 1993; Malenka and Bear, 2004). Homeostatic plasticity, in contrast, occurs more slowly and is not input specific but instead responds to increases or decreases in a neuron’s firing rate to globally and multiplicatively scale synaptic weights, down or up respectively, to maintain a set firing rate (Turrigiano and Nelson, 2004). Homeostatic plasticity is an important mechanism by which synaptic weights are renormalized, counteracting through synaptic scaling the runaway dynamics of homosynaptic plasticity, which would otherwise saturate the capacity of neurons to undergo further change. However, it should be noted that the term homeostatic plasticity has been applied to several, distinct types of plasticity, the details of which are beyond the scope of this review (discussed in more detail by Fox and Stryker, 2017, and the references therein).

Homosynaptic and homeostatic plasticity are the two forms of plasticity most frequently proposed to explain the majority of observed developmental circuit refinement. However, bridging the divide between homosynaptic and homeostatic plasticity are forms of heterosynaptic plasticity that can act in concert with or opposition to homosynaptic changes by altering the structure and strength of neighboring synapses. Although heterosynaptic plasticity was discovered soon after homosynaptic plasticity (Lynch et al., 1977), there is surprisingly little known about how heterosynaptic mechanisms act to regulate the development of neuronal circuits. This lack is partially due to the challenge in distinguishing homosynaptic from heterosynaptic mechanisms, as this requires the identification of the specific synapses activated by a plasticity-inducing manipulation. Additionally, both homeostatic and heterosynaptic plasticity can lead to renormalization of synaptic weights, albeit on different spatial and temporal scales and with different functional consequences. While homeostatic plasticity is thought to occur on slower timescales and involves cell-wide synaptic scaling to globally renormalize homosynaptic increases or decreases in strength, heterosynaptic plasticity can occur on faster timescales to renormalize synaptic strength on local stretches of dendrites, while reinforcing homosynaptic plasticity in a locally coordinated manner. With the development of new tools and techniques to functionally and structurally measure and manipulate synapses *in vivo*, it is now possible to critically examine the role of heterosynaptic plasticity in neuronal development.

**Figure 1:**
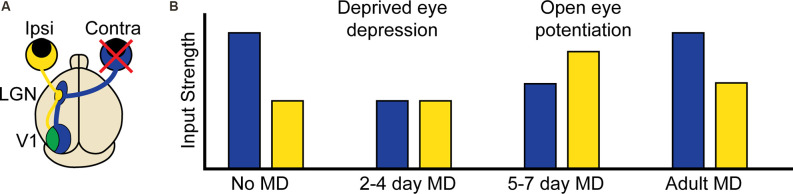
Ocular dominance plasticity in the mouse binocular visual cortex. **(A)** Visual information from the contralateral (contra, blue) eye first arrives in the contralateral lateral geniculate nucleus (LGN) of the thalamus. Visual information corresponding to the binocular portion of the visual field also arrives from the ipsilateral (ipsi, yellow) eye, but remains largely segregated. This information is then relayed to the visual cortex (V1), where contralateral and ipsilateral visual input corresponding to the binocular portion of the visual field converge in the binocular region. **(B)** At baseline, the contralateral eye input to binocular V1 is approximately twice as strong as the ipsilateral input. In critical period aged mice, 2–4 days of contralateral monocular deprivation (MD, red X) results in the depression of deprived eye (contralateral) input. At 5–7 days post MD, there is a potentiation of open eye (ipsilateral) input. In adult mice following MD, contralateral depression does not occur but there is still open eye potentiation.

Experience-dependent plasticity is, perhaps, best understood in the development of the binocular region of the primary visual cortex (binocular V1; Figure 1A), and in particular the experience-dependent process of matching the input from the two eyes onto the same postsynaptic neurons (Espinosa and Stryker, 2012; Levelt and Hübener, 2012; Hooks and Chen, 2020). Disrupting patterned vision through one eye [monocular deprivation (MD)] during a developmental critical period leads to a competitive loss of input from the deprived eye and a delayed increase in input from the open eye in a process termed ocular dominance plasticity (ODP; Figure 1B). The direct translatability of ODP to the loss of visual acuity observed in amblyopia, ease of induction, and ease of measurement combine to make ODP one of the most studied form of developmental experience-dependent plasticity in the cortex. This tractability has allowed scientists to identify key molecules, implicated in both homosynaptic and homeostatic plasticity, required for or interfering with the expression of experience-dependent plasticity. Despite these findings, it is still unclear what synaptic changes underlie the distinct phases of ODP, and if the shared molecular requirements equate to the sufficiency of homosynaptic and homeostatic mechanisms to explain them.

The functional response changes that characterize ODP are thought to involve “feedforward” plasticity which leads to reduction of deprived eye responses in binocular V1 neurons, and “feedback” plasticity which leads to enhancement of open-eye responses (Figure 1; Tropea et al., 2009b). We propose that heterosynaptic plasticity contributes to both feedforward and feedback plasticity during ODP and that the inclusion of heterosynaptic plasticity alongside homosynaptic and homeostatic mechanisms can resolve conflicting experimental findings and provide a richer mechanistic understanding of experience-dependent developmental plasticity. In this review, we will first define the types of observed plasticity classified as heterosynaptic, the molecular mechanisms that drive heterosynaptic changes, and how such processes are thought to contribute to shaping neuronal output. We will then summarize recent findings that strongly suggest heterosynaptic mechanisms are at work *in vivo*, as well as the overlap between the molecular pathways implicated in both heterosynaptic plasticity and ODP. Finally, we will propose functions for heterosynaptic plasticity *in vivo* that make testable predictions for future investigation.

## What Is Heterosynaptic Plasticity?

### Compensation

Heterosynaptic plasticity was first described in the CA1 region of the rat hippocampus (Lynch et al., 1977), where the anatomical segregation of distinct inputs is amenable to probing questions of input specificity. They separately recorded two distinct input pathways and found that high-frequency stimulation of one pathway led to its potentiation, as expected from homosynaptic plasticity, but also led to the depression of the second, unstimulated pathway, in what was termed heterosynaptic depression (H-LTD). The depression of the unstimulated pathway may serve a homeostatic role in compensating for the potentiation of the stimulated pathway. However, it is distinct from homeostatic plasticity as the depression arises through LTP induction rather than a change in postsynaptic firing rate, occurs specifically for the unstimulated pathway, and occurs rapidly on a similar timescale with homosynaptic potentiation. Compensatory heterosynaptic plasticity occurring alongside homosynaptic plasticity has since been observed between inputs synapsing onto the same neuron (Royer and Paré, 2003; Bian et al., 2015; Oh et al., 2015; Jungenitz et al., 2018; Field et al., 2020; Mendes et al., 2020; Tong et al., 2021), and can also occur following homosynaptic LTD with compensatory heterosynaptic potentiation (H-LTP; Royer and Paré, 2003; Field et al., 2020). The change in the weight of inactive synapses does not always occur in the opposite direction of homosynaptic change, but can instead be dependent on the initial synaptic weight of the inactive synapse, with strong inactive inputs depressed and weak inactive synapses potentiated or left unchanged (Letellier et al., 2016; Bannon et al., 2017; Chistiakova et al., 2019; Field et al., 2020). In some cases, similar to homeostatic plasticity, these weight-dependent changes can occur in the absence of homosynaptic plasticity through postsynaptic spiking alone (Chen J. Y. et al., 2013). Compensatory heterosynaptic plasticity may serve to renormalize neuronal output and prevent runaway homosynaptic dynamics in a non-global and non-multiplicative fashion, but could also provide a means by which to amplify differences between synapses encoding distinct or opposing inputs.

### Facilitation

Rather than acting on inactive synapses, other forms of heterosynaptic plasticity serve to facilitate plasticity in inputs that are active following homosynaptic change. In CA1, three stimulus trains of 100 pulses at 100 Hz lead to late (lasting at least 8 h), protein synthesis-dependent LTP, while a single train leads to early (lasting only 3–5 h), protein synthesis independent LTP (Frey and Morris, 1997). However, prior induction of late LTP in one pathway facilitates the induction of late LTP in a second pathway receiving only a single train. The facilitation of late LTP in the second pathway is credited to a “synaptic tag” produced by either form of LTP that captures proteins made in response to late LTP induction. Such a tag is required for LTP-related proteins produced in the soma to find the potentiated synapses in the dendrites. Similar facilitation occurs at the level of single postsynaptic dendritic spines, where LTP induced at one spine permits LTP induction with a subthreshold stimulus on another spine within a window of 10–90 min (Harvey and Svoboda, 2007; Govindarajan et al., 2011). Facilitative heterosynaptic plasticity may serve to open temporal windows of heightened plasticity following an initial homosynaptic event.

### Cooperation

While a single subthreshold stimulus may be insufficient to drive LTP without facilitative plasticity, if two or more inputs are activated simultaneously by the same subthreshold stimulation, they can, under certain circumstances, all undergo synaptic strengthening (White et al., 1990). This cooperative induction of plasticity seems not to rely on integrative summation in the soma but instead on nonlinear integration within the dendrites themselves (Lee et al., 2016; Weber et al., 2016). Surprisingly, these interactions between simultaneously stimulated inputs can even alter classical spike timing-dependent plasticity rules, widening the allowable temporal window between presynaptic and postsynaptic spiking resulting in LTP, and even preventing LTD when postsynaptic precedes presynaptic spiking (Tazerart et al., 2020). Thus, cooperative heterosynaptic plasticity could serve as a coincidence detector for stimuli occurring close together in time, and by strengthening the active inputs, their coincident activity can more effectively drive action potential firing in the future. While other forms of heterosynaptic plasticity exist between neurons, in this review we will restrict our discussion to the plasticity of synapses on the same postsynaptic neuron (Bonhoeffer et al., 1989; Kossel et al., 1990; Schuman et al., 1994).

### Distance Dependence

In the dentate gyrus, which also has anatomical separation of inputs similar to CA1, the extent of H-LTD in an unstimulated pathway following LTP induction in another pathway correlates with the extent of spatial overlap between them, with no plasticity seen with <50% overlap (White et al., 1990). Additionally, activation of two pathways with subthreshold stimulation could only induce cooperative H-LTP if the pathways overlapped >50%. These findings were an early indication, borne out by later work, that the physical distance between synapses is a crucial factor for heterosynaptic plasticity, and suggests that heterosynaptic plasticity is mediated by a diffusible factor or electrical conductance. However, it is methodologically challenging to locally stimulate one synapse or even a group of synapses without also stimulating its neighbors. An early attempt to overcome this relied on silencing activity in an organotypic hippocampal slice using a solution of cadmium and low calcium (Ca^2+^), and then superfusing a small area (~30 μm) with normal culture medium and elevated Ca^2+^ to restore local activity. The authors found that groups of synapses within 70 μm of the site of homosynaptic LTP induction are also potentiated (despite being silenced) suggesting a breakdown of homosynaptic plasticity at short distances (Engert and Bonhoeffer, 1997). Although not credited to heterosynaptic plasticity, this spread of LTP from the stimulated region to adjacent regions could conceivably arise through facilitation from the test pulses, or cooperative activation by incomplete silencing near the site of superfusion. A way to probe synaptic specificity with better spatial restriction was clearly needed to advance the field.

**Figure 2:**
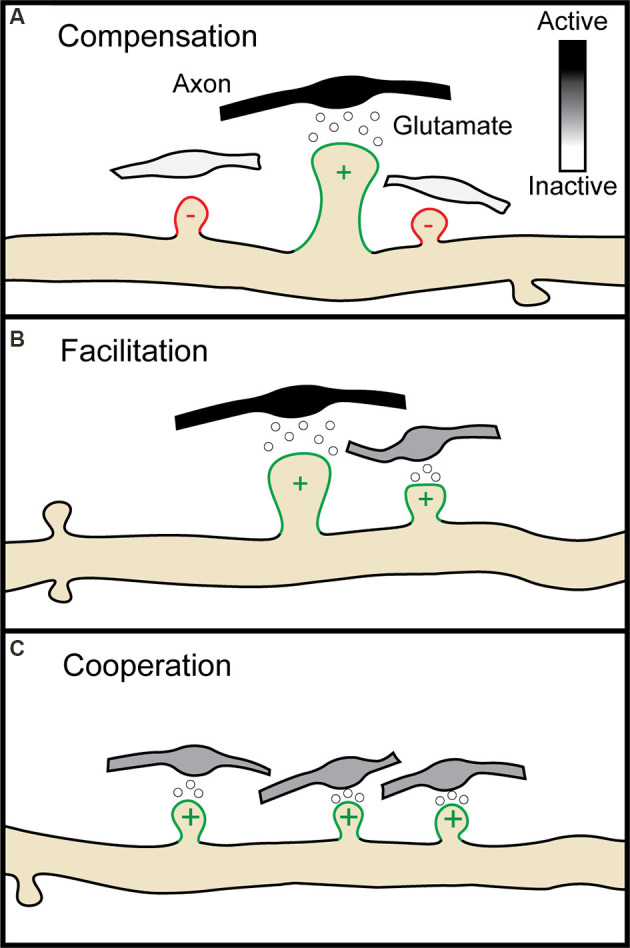
Multiple forms of heterosynaptic plasticity. **(A)** Homosynaptic potentiation at a single synapse (green spine, +), represented here by the release of glutamate from an active presynaptic axon (black boutons), results in compensatory heterosynaptic depression of nearby inactive synapses (white boutons, red spines, −). **(B)** Homosynaptic potentiation at one spine can facilitate later potentiation at a neighboring spine receiving a subthreshold stimulus (gray bouton). **(C)** Four neighboring spines receiving subthreshold stimulation undergo potentiation through cooperative heterosynaptic plasticity.

Around the same time, a new technique was developed which relied not on physical probes but on inert, or so-called “caged” forms of neurotransmitters that could be rendered chemically active, or “uncaged”, with single synapse precision through the use of one and two-photon laser excitation (Callaway and Katz, 1993; Denk, 1994). With these tools in hand, scientists could begin to probe the spatial constraints of multiple forms of heterosynaptic plasticity. LTP induction at single spines with glutamate uncaging indeed induces compensatory shrinkage of neighboring spines within roughly 3 μm (Figure 2A ; Oh et al., 2015; Tong et al., 2021), while spines within 3–8 μm are instead potentiated (Tong et al., 2021). Early LTP at single spines can facilitate early H-LTP at neighboring spines within 8 μm for 10 min (Figure 2B; Harvey and Svoboda, 2007; Hedrick et al., 2016), while late LTP can facilitate late H-LTP at neighboring spines within 70 μm for upwards of 45 min (Govindarajan et al., 2011). Cooperative H-LTP of 2–4 spines requires the spines to be clustered together within 3–10 μm of one another (Figure 2C; Lee et al., 2016; Weber et al., 2016; Magó et al., 2020; Tazerart et al., 2020). Other, less common forms of heterosynaptic plasticity also occur locally in adjacent spines. *In vivo*, induction of LTD at single spines through uncaging leads to the spread of H-LTD to other spines within 3 μm (Noguchi et al., 2019), while driving LTD at two spines with glutamate uncaging fails if the spines are within 40 μm of one another (Tazerart et al., 2020). The breakdown of homosynaptic plasticity by heterosynaptic interactions thus appears to be driven in large part by the locally coordinated activity of neighboring synapses. In the following section, we will describe the molecular mechanisms that make such spatial specificity possible. We will separately discuss the molecules involved in the induction of plasticity and those involved in the spread of plasticity, or crosstalk, between neighboring synapses.

## Molecular Mechanisms of Heterosynaptic Plasticity

### Induction

NMDA receptors function as coincidence detectors for glutamate binding and postsynaptic depolarization due to their voltage-dependent blockage by magnesium. This property makes NMDA receptors central to the input specificity of homosynaptic plasticity (Collingridge et al., 1983). Given their close relationship, it is perhaps unsurprising that this crucial receptor is also indispensable for multiple forms of heterosynaptic plasticity. In hippocampal CA3, activation of NMDA receptors by mossy fiber inputs triggers H-LTP of perforant path inputs (Tsukamoto et al., 2003). In the dentate gyrus, LTP of the middle molecular layer leads to compensatory H-LTD of the outer molecular layer, and both homo- and heterosynaptic changes are dependent on NMDA receptors (Jungenitz et al., 2018). In the subthalamic nucleus, NMDA receptors also regulate the heterosynaptic strengthening of inhibitory inputs, highlighting that heterosynaptic plasticity can also occur through changes in inhibitory connections (Chu et al., 2015). The role of NMDA receptors in heterosynaptic plasticity is not necessarily restricted to the postsynaptic neuron, or indeed to neurons at all. Heterosynaptic plasticity can occur presynaptically to balance input from two neurons onto the same postsynaptic cell, and this process was found to require NMDA receptor activation on astrocytes (Letellier et al., 2016). At the local level, distance-dependent compensatory and cooperative plasticity, as well as functional clustering of groups of coactive spines, also depend on NMDA receptor activity (Kleindienst et al., 2011; Takahashi et al., 2012; Niculescu et al., 2018; Letellier et al., 2019; Magó et al., 2020). Is the loss of heterosynaptic plasticity following NMDA receptor blockade simply due to the role of NMDA receptors in homosynaptic plasticity, or are NMDA receptors separably required for both types of plasticity? As discussed in more detail below, we now know that Ca^2+^ entry through NMDA receptors, and the host of molecules activated downstream of NMDA receptor signaling, can spread from an activated dendritic spine to its neighbors (Rose et al., 2009; Chu et al., 2015; Hedrick et al., 2016; Lee et al., 2016; Jungenitz et al., 2018; Niculescu et al., 2018; Tazerart et al., 2020), making NMDA receptor activation central to both homo- and heterosynaptic forms of plasticity.

Other external factors can also act to induce or regulate heterosynaptic plasticity between synapses. In particular, BDNF signaling through TrkB receptors is crucial in the cooperative stabilization of coactive clusters of dendritic spines (Niculescu et al., 2018), and is required at the neighboring spine receiving subthreshold input during facilitatory H-LTP (Hedrick et al., 2016). Conversion of proBDNF to BDNF by MMP9 is required for this facilitation, and intriguingly proBDNF itself, downstream of NMDA receptor activation, can lead to H-LTD through p75NTR activation (Niculescu et al., 2018). Besides acting through NMDA receptors, glutamate signaling through mGluRs is required for some forms of heterosynaptic plasticity (Oh et al., 2015), but not others (Chu et al., 2015; Lee et al., 2016). Adenosine does not appear to directly contribute to heterosynaptic plasticity, but increasing the concentration of adenosine eliminates the weight dependence of heterosynaptic plasticity following LTP (Bannon et al., 2017). Since adenosine release is brain state-dependent, increasing after periods of activity or during sleep, this allows synaptic plasticity to shift from behaving homosynaptically to heterosynaptically in a state-dependent manner. Outside of receptor signaling, the cadherin-catenin cell adhesion complex is known to be a critical component for synaptic stabilization, but can also signal heterosynaptically to depress or eliminate neighboring synapses (Bian et al., 2015). Enhancing the function of the neural cell adhesion molecule NCAM can permit H-LTP in the dentate gyrus of the unstimulated lateral perforant path following LTP induction at the medial perforant path (Dallérac et al., 2011). Thus, factors with known roles in homosynaptic plasticity can also function to induce heterosynaptic effects.

### Crosstalk

Several signaling pathways downstream of NMDA and TrkB receptor activation are crucial for heterosynaptic signaling. In particular, activation of Ca^2+^-calmodulin-dependent protein kinase II (CaMKII) by Ca^2+^ entry through NMDA receptors is required for H-LTP, but not H-LTD with CaMKII blockade, in fact, biasing heterosynaptic plasticity towards H-LTD (Chu et al., 2015; Oh et al., 2015; Tong et al., 2021). In contrast, Ca^2+^-dependent activation of the serine/threonine protein phosphatase calcineurin is required for H-LTD, and blockage of calcineurin leads to H-LTP of spines that would otherwise undergo H-LTD (Oh et al., 2015; Tong et al., 2021). Ca^2+^ induced Ca^2+^ release from the endoplasmic reticulum can also be required for heterosynaptic plasticity downstream of NMDA receptors (Nishiyama et al., 2000; Royer and Paré, 2003; Oh et al., 2015; Lee et al., 2016), particularly during development when Ca^2+^ can propagate several μms along the dendrite, whereas in adulthood Ca^2+^ spread is restricted to the activated spine (Lee et al., 2016). The effectiveness of Ca^2+^ induced Ca^2+^ release *via* NMDA receptors is likely dependent on the proximity of the endoplasmic reticulum, as clusters of spines following LTP induction are more likely to occur near a spine containing ribosomes and smooth endoplasmic reticulum (Chu et al., 2015). Thus, in adulthood when the spatial spread of Ca^2+^ is more confined, heterosynaptic plasticity may only be permissible near select, privileged spines that can propagate Ca^2+^ dependent signaling. Ca^2+^ entry through NMDA receptors may itself be important to the propagation of heterosynaptic plasticity by initiating dendritic NMDA receptor-dependent depolarizing spikes (Schiller et al., 2000; Losonczy and Magee, 2006), which can arise from cooperative activation of clustered dendritic spines and lead to H-LTP independently of postsynaptic action potentials (Magó et al., 2020). However, the effectiveness of NMDA receptor mediated Ca^2+^ influx in initiating spikes, as well as the rules governing NMDA receptor-dependent plasticity, can vary between proximal and distal dendrites (Gordon et al., 2006; Weber et al., 2016). In contrast to NMDA receptors, Ca^2+^ entry through voltage-gated Ca^2+^ channels does not appear to be critical for the propagation of heterosynaptic plasticity (Lee et al., 2016; Weber et al., 2016; Magó et al., 2020). However, voltage-gated Ca^2+^ channels are required on astrocytes for their role in heterosynaptically regulating presynaptic strength (Letellier et al., 2016).

The local nature of heterosynaptic plasticity strongly suggests that a molecular factor or factors spread from the site of initiation to invade neighboring dendritic spines, altering their strength or propensity for further plasticity. Which factors downstream of induction remain confined to the activated synapse, and which factors diffuse outward? To address this question, Harvey et al. (2008) used a FRET-based indicator of H-Ras activation, a GTPase that lies downstream of NMDA receptor and CaMKII-dependent LTP. Using glutamate uncaging at single spines to induce LTP, they found that far from being restricted to the potentiated spine, activated H-Ras spreads over 11 μm along the dendrite and invades neighboring spines. While activated H-Ras is necessary for both homosynaptic potentiation and facilitatory H-LTP, disrupting H-Ras signaling to ERK only impairs heterosynaptic facilitation. Thus, the homosynaptic and heterosynaptic functions of H-Ras can be disambiguated through live-cell imaging and manipulation of protein dynamics. A similar strategy was later used to study two other GTPases downstream of CamKII; Cdc42 and RhoA (Murakoshi et al., 2011). While activated Cdc42 remains confined to the potentiated spine, activated RhoA diffuses laterally up to 10 μm to invade neighboring spines, although this invasion is by itself insufficient to initiate heterosynaptic plasticity. A landmark study by Hedrick et al. (2016), expanded upon this study, demonstrating that the spread of activated Rac1 and RhoA through the dendritic shaft is necessary for H-LTP, and that Cdc42 activation, but not Rac1 and RhoA, can be induced by subthreshold stimulation. Thus, H-LTP requires the diffusion of activated H-Ras, Rac1, and RhoA from the initially potentiated spine, and the activation of Cdc42 in the neighboring spine by a subthreshold stimulus. Further, while activation of all four GTPases is dependent on NMDA receptors and CaMKII; Rac1 and Cdc42 activation are also dependent on BDNF, and partial BDNF inhibition prevents the spread of activated Rac1 and H-LTP. NMDA receptors and BDNF are, therefore, both linked to facilitatory H-LTP through the coregulation of GTPases.

**Figure 3:**
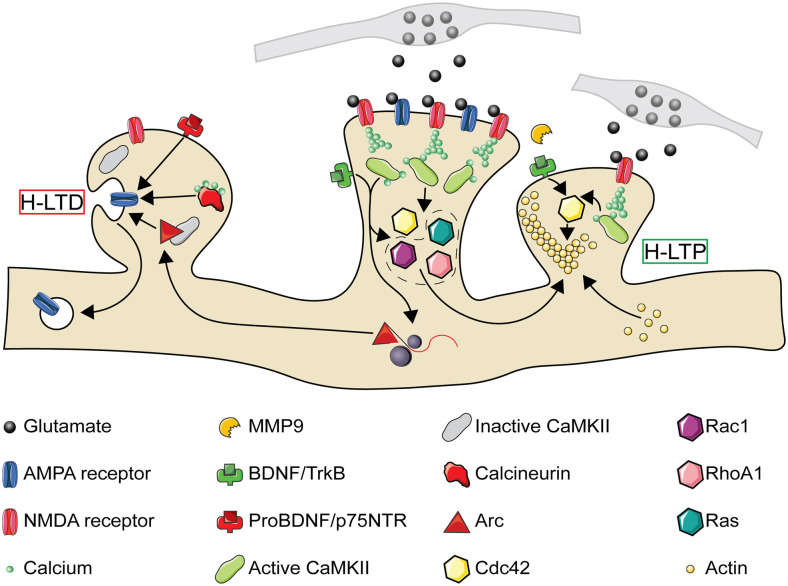
The molecular pathways of heterosynaptic potentiation and depression. A Simplified representation of the molecular pathways through which homosynaptic potentiation (center spine) can drive heterosynaptic depression (H-LTD, left spine) and heterosynaptic potentiation (H-LTP, right spine) of neighboring synaptic spines. At the central spine, presynaptic glutamate release activates postsynaptic AMPA and NMDA receptors. NMDA receptor activation leads to calcium entry into the synapse, which in combination with calmodulin leads to CaMKII activation. CaMKII activation results in the activation of Ras and RhoA1, and in combination with TrkB activation through BDNF also the activation of Cdc42 and Rac1. Both CaMKII and BDNF activation may also result in the local translation of *Arc* mRNA present from previous bouts of activity. While activated Cdc42 remains confined to the activated spine, Ras, RhoA1, Rac1, and Arc spread along the dendritic shaft with the potential to interact with neighboring spines. If a nearby spine is inactive (left), Arc is recruited to the spine by an interaction with inactive CaMKIIβ. Small influxes of calcium, insufficient to activate CaMKII, can activate calcineurin. Release of proBDNF by the activated spine, in the absence of MMP9, can also result in binding of proBDNF to p75 neurotrophin receptors (p75NTR). Each of these processes can promote either structural spine shrinkage or the endocytosis of surface AMPA receptors, leading to H-LTD. If a neighboring synapse is instead activated (right, activation either simultaneous with or following the center spine), MMP9 promotes cleavage of proBDNF to BDNF which binds to TrkB receptors. This, in combination with NMDA receptor driven CaMKII activation, leads to Cdc42 activation. Cdc42 activation by a subthreshold stimulus, in combination with the spread of activated Ras, RhoA1, and Rac1 from the center synapse, drives the remodeling of the actin cytoskeleton leading to structural H-LTP. CaMKII, Ca^2+^-calmodulin-dependent protein kinase II; MMP9, matrix metalloproteinase 9. Image assets reproduced from smart.servier.com (Servier Medical Art, 2015) (CC-BY).

How then might LTP lead to compensatory H-LTD of neighboring inactive synapses? The immediate early gene, *Arc*, is necessary for both LTD and homeostatic downscaling of AMPA type glutamate receptors through Arc protein’s interactions with the endocytic machinery (Lyford et al., 1995; Chowdhury et al., 2006; Plath et al., 2006; Shepherd et al., 2006; Waung et al., 2008). Similar to homosynaptic and homeostatic plasticity, AMPA receptor internalization and insertion are crucial for the expression of H-LTD and H-LTP respectively (Oh et al., 2015; Tazerart et al., 2020). Paradoxically for a protein linked to synaptic weakening, *Arc* mRNA localizes to active dendrites where it is translated in response to further bouts of activity (Steward et al., 1998; Jakkamsetti et al., 2013). This suggests that newly translated Arc protein is well-positioned to mediate H-LTD of inactive synapses in the vicinity of recently potentiated spines. Indeed, after LTP induction by BDNF treatment, newly translated Arc protein localizes to inactive dendritic spines by selectively binding the inactive form of CaMKIIβ (Okuno et al., 2012), and *in vivo* the loss of Arc does, in fact, disrupt H-LTD (El-Boustani et al., 2018). Although Arc is required for mGluR and not NMDA receptor-dependent LTD in the hippocampus (Park et al., 2008; Waung et al., 2008; Jakkamsetti et al., 2013; Wilkerson et al., 2014; but see Plath et al., 2006), the signaling factors upstream of Arc’s role in H-LTD remain unclear. Another potential mechanism for *Arc*’s heterosynaptic spread during plasticity is the newly described role of Arc in forming capsids for intercellular transport of mRNA (Pastuzyn et al., 2018). Postsynaptic release of nitric oxide following LTP leads to heterosynaptic depression of nearby presynaptic release sites within 4 μm (Tong et al., 2021). Similarly, the extracellular spread of Arc may promote local depression of nearby synapses. To summarize, NMDA receptor activation and BDNF regulate not only homosynaptic changes in synaptic strength but heterosynaptic changes through the intracellular and intercellular diffusion of plasticity-related molecules (summarized in Figure 3).

## Implications of Heterosynaptic Plasticity

How does heterosynaptic plasticity impact computations at the neuronal level, and how might this be important for the development of perception and cognition? In this section, we will discuss how heterosynaptic plasticity can alter the fine-scale arrangement of synaptic inputs and how these synaptic modifications can shape neuronal response selectivity. We will also describe the implications of heterosynaptic plasticity for balancing the total synaptic weights of inputs through its role in compensatory plasticity.

### Functional Clustering

Facilitatory and cooperative forms of heterosynaptic plasticity may have a central role in spatially organizing temporally correlated synaptic inputs, a phenomenon known as functional clustering. Two studies (Kleindienst et al., 2011; Takahashi et al., 2012) were among the first to characterize the emergence of functional clustering in developing hippocampal dendrites. Both studies found that co-active synaptic pairs are more likely to emerge if they are less than 16 μm apart and that the organization of these co-active pairs arises through activity-dependent processes since the presence of TTX or NMDA receptor antagonists abolishes the clustering. The mechanisms behind functional clustering are linked to LTP, as half of the spines strengthened by LTP have new, functional spines that emerge in close proximity to them (De Roo et al., 2008). These hotspots for functional clustering appear to require experience, as in the barrel cortex deprivation through whisker trimming has been found to block the cooperative potentiation of clustered spines (Makino and Malinow, 2011).

**Figure 4:**
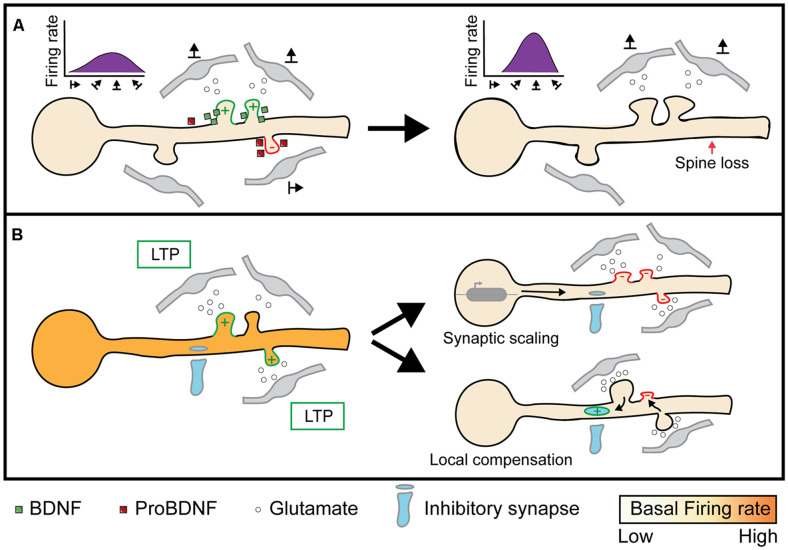
Implications of heterosynaptic plasticity on neuronal activity. **(A)** Facilitatory or cooperative heterosynaptic mechanisms of plasticity stabilizes the formation of functionally clustered synapses and eliminates out-of-phase synapses through BDNF and proBDNF respectively (left; Niculescu et al., 2018). Convergent activity by functional clusters evokes dendritic spikes, or “hotspots”, through NMDA and Na+ mediated currents, which propagates to the soma and initiates neuronal firing. As a result, functional clusters can narrow the range of somatic firing to specific inputs, such as select orientation preferences (right; Wilson et al., 2016). **(B)** When neuronal spiking activity is below or above a specific threshold, homeostatic plasticity is triggered to bring the firing rate back to a “baseline” state. Synaptic scaling is one mechanism that scales all synapses multiplicatively to conserve the total synaptic weight (Lambo and Turrigiano, 2013). On the other hand, compensatory homeostatic plasticity acts on neighboring excitatory, as well as inhibitory, synapses to locally conserve synaptic weights (Royer and Paré, 2003; Oh et al., 2015; Field et al., 2020). Image assets reproduced from smart.servier.com (Servier Medical Art, 2015) (CC-BY).

Several modeling studies have made predictions on the mechanisms that underlie the formation of functional clusters (Legenstein and Maass, 2011; Limbacher and Legenstein, 2020). Kirchner and Gjorgjieva (2021) successfully recapitulated previous experimental findings on the functional clustering of excitatory synapses in visual cortical areas by using a heterosynaptic plasticity model based on activity-dependent interactions between BDNF and proBDNF (Winnubst et al., 2015). The authors found that this introduced a distance-dependent and timing-dependent competition between synapses. Thus, functional clustering can putatively occur through heterosynaptic mechanisms (Figure 4A; Niculescu et al., 2018). At low synaptic densities, heterosynaptic interactions between synapses are minimal, and thus synaptic plasticity is mainly homosynaptic. At high synaptic densities, heterosynaptic interactions increase near homosynaptic sites with neighboring unstimulated synapses weakening and neighboring co-active synapses strengthening. Intriguingly, when the authors also included inhibitory synapses, they found that inhibitory synapses cluster around excitatory synapses if they exhibit anti-correlated response preferences. Thus, a heterosynaptic plasticity regime is sufficient to form and stabilize correlated, clustered inputs.

### Non-linear Dendritic Integration

Functional synaptic clusters can be computationally important for transitioning dendritic branches from passively to actively propagating signals. While the membrane potential of a dendritic branch has classically been assumed to linearly summate active synaptic inputs (Cash and Yuste, 1998, 1999) that decay as a function of distance based on passive cable properties (Rall, 1967), dendritic branches can produce regenerative events, called dendritic spikes, that are evoked through non-linear input integration (Mel, 1993; Johnston et al., 1996). Our understanding of what evokes dendritic spikes has been advanced by several studies that explored the constraints of non-linear integration. For example, Gasparini and Magee (2006) performed simultaneous glutamate uncaging and dendritic patch recordings in CA1 neurons to demonstrate that delivering near synchronous inputs (3 ms apart) within a 20 μm stretch of a distal dendritic branch elicits non-linear increases in dendritic EPSPs. The rise in dendritic voltage potential is smaller and is linearly summed when inputs are delivered asynchronously (10 ms apart) or are distributed across a larger dendritic area (~150–200 μm). Thus, dendritic branches can experience nonlinear transitions in their membrane properties based on the distance and timing of their synaptic inputs.

The contribution of spatial clustering to dendritic spiking activity, however, is also dependent on the distance of the dendritic branch from the soma. A follow-up study examining dendritic integration in oblique, rather than distal, dendrites from CA1 neurons found that dendritic spikes on oblique dendrites require synchronous but not necessarily spatially adjacent inputs (Losonczy and Magee, 2006). The distance-dependent influence of spatial clusters on spiking is currently thought to arise from differences in local biophysical dendritic properties, such as the impedance gradient that increases from the soma to distal branches (Harnett et al., 2012) or the concentration of A-type potassium channels, which temporally constrain NMDA receptor driven Ca^2+^ spikes and also vary in concentration with distance from the soma (Hoffman et al., 1997; Losonczy and Magee, 2006). By simultaneously performing two-photon glutamate uncaging and measuring spine Ca^2+^ responses across the dendritic tree in CA1 neurons, Weber et al. (2016) confirmed that the threshold for evoking non-linear increases in dendritic Ca^2+^ by spatially clustered inputs (spines 3–6 um apart) decreases from proximal (12 coactive spines required) to distal (four coactive spines required) points on dendritic branches. The impact of spatial clusters is also dependent on cell type and input connectivity differences, as layer 4 neurons in the barrel cortex do not exhibit nonlinear increases in spine Ca^2+^ responses during co-activation of neighboring spines (Jia et al., 2014). By forming functional synaptic clusters, heterosynaptic plasticity can directly affect dendritic computation and therefore bias the selectivity of somatic responses towards a few active dendritic branches (Figure 4A). Indeed, V1 neurons are orientation selective (Hubel and Wiesel, 1959), and dendritic spikes share the soma’s orientation preference, sharpen somatic selectivity, and occur in dendritic branches that contain functionally clustered synapses (Smith et al., 2013; Wilson et al., 2016). It will be crucial for future research to identify other functional responses that display such clustering, chart the emergence of clustering over development, and identify by what molecular mechanisms clustering occurs.

### Regulation of Local Homeostasis

Similar to homeostatic plasticity, compensatory heterosynaptic plasticity can balance synaptic weights and prevent runaway dynamics. The phenomena of runaway homosynaptic dynamics has long been appreciated (Bienenstock et al., 1982) and extensively studied in neural networks modeling learning and development (Hasselmo, 1994). To globally balance synaptic strength while ensuring the relative strength between inputs remains stable, computational models commonly scale synapses by the sum of all synaptic weights. While including a renormalization term is computationally effective, it is unclear how synapses could similarly calculate the strength of a neuron’s total synaptic pool and change accordingly (Carlson et al., 2013). Local conservation of synaptic strength, on the other hand, is easily explained through the molecular mechanisms of compensatory heterosynaptic plasticity. To demonstrate that heterosynaptic plasticity can effectively block runaway synaptic dynamics, Chen J. Y. et al. (2013) combined homosynaptic spike timing-dependent plasticity with heterosynaptic plasticity in a multicompartmental model of a layer 2/3 neuron. They found that while synaptic weights tend to stabilize around an equilibrium point, inputs that are highly correlated with the postsynaptic neurons’ output are still stronger than those that are weakly correlated. These results suggest that heterosynaptic plasticity does not obstruct homosynaptic LTP, despite acting only locally to balance synaptic weights. Heterosynaptic plasticity can also mediate renormalization by modifying inhibitory synaptic inputs. Combining whole-cell recordings in layer 5 pyramidal neurons with paired stimulation from a multi-channel electrode, Field et al. (2020) demonstrated that both unstimulated excitatory and inhibitory inputs undergo plasticity to modify the correlation between excitatory and inhibitory input strengths. Thus, heterosynaptic plasticity can regulate the intrinsic excitability of neurons by: (1) renormalizing the strength of excitatory synapses and (2) maintaining excitatory-inhibitory balance at a local level (Figure 4B). While developmental pruning of excitatory synapses and maturation of inhibitory input are both crucial to neuronal development (Levelt and Hübener, 2012; Faust et al., 2021), it is unclear as to what degree heterosynaptic mechanisms contribute to these processes.

## *In Vivo* Plasticity-Mediated Synaptic Reorganization on Dendrites

### Functional Organization of Synaptic Inputs

Advancements in Ca^2+^ indicators have made it possible to record Ca^2+^ signals within single dendritic spines *in vivo*, permitting the mapping of functional synaptic inputs onto dendrites and quantification of their degree of clustering (Chen et al., 2011; Chen T.-W. et al., 2013; Winnubst et al., 2015; Gökçe et al., 2016; Wilson et al., 2016; Iacaruso et al., 2017; Scholl et al., 2017, 2021; El-Boustani et al., 2018; Kerlin et al., 2019; Lee et al., 2019; Ju et al., 2020). The presence of functional synaptic clustering in these studies strongly indicates that experience-dependent synaptic plasticity involves heterosynaptic interactions. Similar to previous observations of dendritic Ca^2+^ spikes, the degree of functional clustering observed varies based on the layer, cell-type, brain state, cortical area, species, or age examined. Due to these differences, we have some insight into the prerequisites for functional clustering. For example, the presence of cortical columns or topographic maps, in which there exists a spatial organization of neurons with similar response properties, can explain differences in functional clustering between species (Kirchner and Gjorgjieva, 2021). In V1, neurons demonstrate functional clustering for orientation preference specifically in species that have orientation columns and small receptive field diameters for visual space, such as macaques and ferrets (Wilson et al., 2016; Ju et al., 2020; Scholl et al., 2021). On the other hand, mice do not have orientation columns and likewise, do not exhibit functional clustering for orientation preference (Iacaruso et al., 2017; Lee et al., 2019). There is, however, evidence for clustering of retinotopic receptive fields in mice (Iacaruso et al., 2017), which can be attributed to the retinotopic organization of mouse V1. These results suggest that global neuronal organization of response properties is a prerequisite for local dendritic clustering of experience-driven responses which, as discussed above, likely emerges through heterosynaptic mechanisms. Without large-scale neuronal organization, the probability of similarly tuned inputs synapsing onto the same dendritic branch within the tight spatial limits of heterosynaptic plasticity may be too low for heterosynaptic plasticity to meaningfully contribute to somatic response selectivity.

### Synaptic Remodeling

The argument for the involvement of heterosynaptic plasticity in synaptic remodeling has been strengthened *in vivo* by the advance of longitudinal two-photon imaging of dendritic spines. Numerous studies performed over development and learning have shown that the rate of dendritic spine turnover and formation is strongly dependent on experience (Trachtenberg et al., 2002; Holtmaat et al., 2005; Zuo et al., 2005a, b; Hofer et al., 2009; Xu et al., 2009; Chen et al., 2012; Fu et al., 2012; Lai et al., 2012; van Versendaal et al., 2012; Yang et al., 2014, 2016; Miquelajauregui et al., 2015; Barnes et al., 2017; El-Boustani et al., 2018; Frank et al., 2018; Jungenitz et al., 2018; Kumar et al., 2020). The experience-dependence of synaptic dynamics is consistent across different brain areas and occurs for both excitatory and inhibitory synapses. Many of these studies also observe that the location of spine formation or elimination occurs non-randomly and is locally-coordinated between neighboring spines, suggesting that heterosynaptic interactions are taking place during the reorganization of spines.

Novel experiences can promote clustered spine formation (Fu et al., 2012; Yang et al., 2014, 2016; Frank et al., 2018; Kumar et al., 2020), and clustered spine elimination can also occur in response to learning, as well as during sensory enrichment and deprivation (Chen et al., 2012; Lai et al., 2012; Frank et al., 2018; Kumar et al., 2020). Some studies have found that clustered spine elimination is related to encoding specific information such as familiar memories (Kumar et al., 2020) or fear conditioning (Lai et al., 2012). Frank et al. (2018) have suggested that experience-dependent spine elimination could facilitate clustered spine formation during learning. Specifically, they found that high basal rates of dendritic spine turnover are strongly predictive of learning a fear conditioning task and are positively correlated with the formation of stable synaptic clusters. Further, these synaptic clusters emerge in the same areas that experienced dendritic spine loss, indicating that spine turnover could provide space for similar presynaptic inputs to form stable synapses through facilitative or cooperative heterosynaptic potentiation. Clustered synaptic turnover can also occur between neighboring inhibitory and excitatory synapses. By expressing fluorescently tagged gephyrin protein, a postsynaptic marker for inhibitory synapses, in YFP-labeled layer 2/3 V1 neurons, Chen et al. (2012) found that inhibitory synapses mirrored the dynamics of neighboring excitatory dendritic spines within 10 μm following MD in adult mice. The authors theorized that this type of clustered excitatory/inhibitory dynamics could explain why ODP is partially reduced in adults compared to critical period age animals.

Although there is substantial evidence supporting cooperative or facilitatory mechanisms of heterosynaptic plasticity *in vivo*, several recent studies in V1 have also demonstrated local compensatory mechanisms (Barnes et al., 2017; El-Boustani et al., 2018). Barnes et al. (2017) characterized structural plasticity of dendritic spines 48 h after monocular enucleation in adult mice and found that only a subset of dendritic branches experienced spine growth. The increase in spine size was correlated with the fraction of spines eliminated in the dendritic branch, indicating that renormalization occurs at the level of dendritic branches rather than neuron wide, which suggests that the mechanism is not homeostatic. El-Boustani et al. (2018) performed a controlled plasticity paradigm where they paired optogenetic stimulation of a layer 2/3 pyramidal neuron in V1 of young mice with the presentation of a visual stimulus at a target location outside of the neuron’s receptive field. Through this optogenetic pairing, they were able to robustly shift the neuron’s receptive field to the target location. The receptive fields of dendritic spines on the neuron were recorded before optogenetic pairing, and the authors found that spines that preferred the target location prior to pairing increase in size after pairing, which is reflective of homosynaptic potentiation and putatively drives the change in the somatic receptive field. However, spines with receptive field preferences away from the target location decrease in size after pairing only if they were in close proximity to a potentiated spine, suggesting that homosynaptic potentiation is coordinated with H-LTD in neighboring spines. The authors found that spines undergoing structural depression are enriched in Arc and have reduced levels of GluA1, highlighting a molecular mechanism shared with the induction of compensatory H-LTD *in vitro*. Intriguingly, knock down of Arc expression prevented not only the distance dependence of spine LTD but the functional shift in the neuron’s receptive field, suggesting that H-LTD is a critical component in altering somatic response properties. This study was the first to demonstrate *in vivo* that heterosynaptic plasticity mechanisms act in a locally coordinated manner to alter neuronal response features, in this case, receptive field location and strength.

To summarize, spine organization and dynamics *in vivo* suggest cooperative or facilitatory forms of heterosynaptic plasticity shape the fine-scale organization of synaptic inputs by clustering neighboring synapses with similar response properties. In addition, the molecular mechanisms of compensatory heterosynaptic plasticity are engaged during activity-dependent shifts in neuronal response preferences. While the majority of these studies were carried out in adult animals, we hypothesize that similar heterosynaptic mechanisms are at work, and potentially heightened, during development. In support of this hypothesis, we will highlight the molecular mediators of heterosynaptic plasticity we have already described that are also implicated in experience-dependent developmental plasticity. Specifically, we will focus on critical period ODP, a form of developmental plasticity whose molecular requirements have been extensively investigated for several decades.

## Developmental Plasticity in Visual Cortex and Heterosynaptic Plasticity

Our progressive understanding of the molecular mechanisms of ODP has been described in detail elsewhere (Tropea et al., 2009b; Levelt and Hübener, 2012; Hooks and Chen, 2020). Here, we will highlight observations where the phenomena and molecular mechanisms of ODP intersect with those of heterosynaptic plasticity (Table 1). During the critical period, ODP following MD takes place through deprived eye depression within 2–4 days, followed by later open eye potentiation typically occurring by 5–7 days post-MD. In adult mice, deprived eye depression is absent or reduced but open eye potentiation still occurs (Figure 1B). Blockade of NMDA receptor signaling pharmacologically or using antisense oligonucleotides against the obligatory NMDA receptor subunit NR1 blocks deprived eye depression following MD during the critical period (Bear et al., 1990; Roberts et al., 1998), and MD occludes later LTD induction (Heynen et al., 2003; Yoon et al., 2009), which in layer 4 (the principal thalamocortical recipient layer) is dependent on NMDA receptor activation and AMPA receptor endocytosis (Crozier et al., 2007). These results fit the model of feedforward LTD of thalamocortical input to layer 4 underlying deprived eye depression. While adult ODP occurs through NMDA receptor dependent open eye potentiation (Sawtell et al., 2003), critical period open eye potentiation requires astrocytic TNFα, which mediates homeostatic scaling of synaptic strength (Kaneko et al., 2008; Ranson et al., 2012) and molecules such as STAT1 (Nagakura et al., 2014) and MVP, a molecule upstream of STAT1 (Ip et al., 2018) that also regulate AMPA receptor insertion that underlies open eye potentiation (Lambo and Turrigiano, 2013). These results suggest that open eye potentiation is mechanistically mediated by feedback homeostatic plasticity. While the blockade of NMDA receptors or TNFα signaling indicates that these mechanisms are separably required for the two phases of ODP to occur, it does not equate to the sufficiency of homosynaptic and homeostatic mechanisms to fully explain the eye-specific shifts during ODP.

**Table 1:** The molecular overlap between heterosynaptic and ocular dominance plasticity.

Molecule	Role in heterosynaptic plasticity	Manipulation	ODP phenotype
NMDA receptors	Induction of compensatory, facilitatory, and cooperative plasticity	APV (antagonist) NR1 antisense oligonucleotide NR1 KO in exc. Cortical neurons NR2B antisense oligonucleotide NR2A KO	No closed eye depression (Bear et al., 1990) No ODP (Roberts et al., 1998) No adult ODP (Sawtell et al., 2003) No ODP in ipsi hemisphere (Cao et al., 2007) No closed eye depression (Cho et al., 2009)
BDNF/TrkB	Induction of facilitatory and cooperative plasticity	Premature BDNF expression in exc. cortical neurons	Premature closure of the ODP critical period (Huang et al., 1999)
ProBDNF/p75NTR	Induction of compensatory plasticity	p75NTR KO in parvalbumin cells	Restores critical period like ODP in adults (Baho et al., 2019)
mGluR	Induction of some forms of compensatory plasticity	mGluR5 heterozygotes Chronic CTEP (negative allosteric modulator) MCPG (antagonist)	No closed eye depression (Dölen et al., 2007) Reduced closed eye depression (Sidorov et al., 2015) No effect on ODP (Hensch and Stryker, 1996)
ERK	Activation by H-Ras required for facilitatory plasticity	U0126/PD98059 (MAPK inhibitors) U0126/CGP57380 during sleep (MAPK/Mnk1 inhibitors)	No ODP (Di Cristo et al., 2001) No ODP (Dumoulin et al., 2015)
CaMKII	Required for facilitatory plasticity	CaMKIIα KO CaMKIIα autophosphorylation deficient mutant	Diminished ODP (Gordon et al., 1996) Diminished ODP (Taha and Stryker, 2005)
Calcineurin	Required for compensatory plasticity	Calcineurin overexpression in exc. cortical neurons	No ODP or critical period closure (Yang et al., 2005)
MMP9	Mediates BDNF induction of facilitatory and cooperative plasticity	GM6001 (MMP inhibitor) MMP9 KO	No open eye potentiation (Spolidoro et al., 2012) Delayed ODP (Kelly et al., 2015)
Voltage gated Ca^2+^ channels	Required on astrocytes for heterosynaptic presynaptic plasticity	TTA-11 (T-type antagonist) Mibefradil (T-type antagonist)	Diminished ODP (Uebele et al., 2009) No open eye potentiation (Yoshimura et al., 2008)
H-Ras	Required for facilitatory plasticity	Constitutive activation	Accelerates open eye potentiation (Kaneko et al., 2010)
Rac1	Required for facilitatory plasticity	CNF1 (inhibits GTP hydrolysis, constitutive activation)	Increased open eye potentiation in adult ODP (Cerri et al., 2011)
Arc	Required for compensatory plasticity	Arc KO Arc overexpression	No ODP (McCurry et al., 2010) Restores critical period like ODP in adults (Jenks et al., 2017)
β-Catenin	Induction of compensatory plasticity	β-Catenin KO in adult exc. Cortical neurons	No effect on adult ODP (Saiepour et al., 2018)
Nitric Oxide	Required for presynaptic compensatory plasticity	L-NMMA/L-NOArg (nitric oxide synthase inhibitors)	No effect on ODP (Ruthazer et al., 1996)
TNFα	Unknown function	TNFα KO	No open eye potentiation (Kaneko et al., 2008)
IGF1	Unknown function	I.P. injection of IGF1	No ODP after 7 days (Tropea et al., 2006)

Indeed, silencing activity in V1 ipsilateral to the deprived eye, acutely or throughout MD, enhances deprived eye responsiveness in the contralateral cortex indicating that callosal inputs contribute to deprived eye depression (Restani et al., 2009). Although mice lack functional clustering of orientation preference, it was recently found that callosal orientation selective synapses cluster near similarly tuned non-callosal synapses (Lee et al., 2019). Thus, callosal synapses are positioned to heterosynaptically interact with thalamocortical synapses. Additionally, a homeostatic explanation of open eye potentiation suggests that both deprived and open eye synapses would be multiplicatively strengthened, however, there are conflicting findings on whether deprived eye potentiation occurs (Gordon et al., 1996; Frenkel and Bear, 2004; Mrsic-Flogel et al., 2007; Lambo and Turrigiano, 2013). Supporting a non-homeostatic mechanism, MD leads to a shift in the NR2 subunits of the NMDA receptor by increasing NR2B production to lower the NR2A/NR2B ratio (Chen and Bear, 2007), which shifts the metaplastic threshold for LTP/LTD induction (Cho et al., 2009). Indeed, KO of NR2A leads not only to the loss of deprived eye depression but also precocious open eye potentiation. These findings collectively suggest that multiple mechanisms underlie the expression of both deprived eye depression and open eye potentiation.

Plasticity-related molecules can have multiple functions in a neuronal circuit, and it can be challenging to isolate which functions are responsible for an observed phenotype in a germline knockout animal or following pharmacological blockade. The difficulty in isolating cell or synapse specific actions of a manipulation is, perhaps, best exemplified by BDNF. Accelerating the onset of BDNF expression in excitatory neurons leads to premature maturation of inhibitory signaling (Huang et al., 1999). The maturation of inhibition is crucial to both the onset and closure of the ODP critical period (Hensch, 2005; Levelt and Hübener, 2012), but this dependence may mask other roles of BDNF in ODP. For instance, infusion of BDNF or blockade of signaling to TrkB both disrupt the formation of OD columns in cats (Cabelli et al., 1995, 1997). This failure in segregation is similar to the observed loss of synaptic clustering with global activation or blockade of BDNF-TrkB signaling in mouse neurons (Hedrick et al., 2016; Niculescu et al., 2018). Local infusion of BDNF in V1 during MD also leads to a paradoxical increase in deprived eye responses, indicating that increased BDNF-TrkB signaling reverses the direction of experience-dependent MD induced synaptic change (Galuske et al., 1996). In the auditory cortex, on the other hand, BDNF infusion amplifies the effects of critical period pure tone exposure (Anomal et al., 2013), exemplifying the region and modality-specific functions of plasticity-related molecules. In contrast to BDNF-TrkB signaling, signaling to p75NTR on parvalbumin interneurons slows the rate of parvalbumin innervation of other neurons, and p75NTR activation on parvalbumin interneurons can restore critical period-like ODP in adult V1 (Baho et al., 2019). It remains to be seen how postsynaptic TrkB and p75NTR signaling on excitatory neurons influence the expression of ODP during the critical period. Future studies could perhaps resolve this question through selective deletion or blockade of receptors in individual neurons or synapses.

While BDNF and its downstream signaling have been extensively studied in both heterosynaptic plasticity and ODP, the function of other growth factors in heterosynaptic plasticity remains unexplored. An unbiased screen for molecules mediating ODP led to the discovery of gene sets and signaling pathways downstream of the growth factor IGF1 as closely involved in ODP (Tropea et al., 2006). Indeed, several molecules regulated by IGF1, including PI3K and phospho-AKT, are downregulated following MD, and application of IGF1, which upregulates these molecules, prevents both deprived eye depression and open-eye potentiation following MD. Interestingly, open eye potentiation is enhanced following the loss of MeCP2, a gene which is mutated in the neurodevelopmental disorder Rett Syndrome (Tropea et al., 2009a), and treatment with IGF1 offsets the abnormality (Castro et al., 2014). In this context, it is worth noting that mouse models of several neurodevelopmental disorders demonstrate deficits in ODP and regulation of overall synaptic strength (Nelson and Valakh, 2015; Mullins et al., 2016). Determining whether these developmental and autoregulatory deficits arise mechanistically from impaired homeostatic or compensatory heterosynaptic plasticity is likely to be important for treating such disorders (Sahin and Sur, 2015).

While the function of MMP9 in converting proBDNF to BDNF remains unexplored in ODP; MMP9 regulates the activity-dependent degradation of the extracellular matrix (Murase et al., 2019), which can restore critical period-like plasticity to adult V1, as maturation of the extracellular matrix around inhibitory parvalbumin interneurons is partially responsible for closing the critical period (Pizzorusso et al., 2002). Extracellular matrix degradation is also a key step in increasing dendritic spine dynamics following MD (Oray et al., 2004). During the critical period, inhibiting MMP activity prevents open eye potentiation and increased spine density in layer 2/3 following MD, but does not block homeostatic plasticity, as observed by measuring increased deprived eye responses in monocular V1 (Spolidoro et al., 2012). Contrary to this, mice with MMP9 knocked out have impaired ODP at 4 but not 7 days post MD, as well as reduced spine density and MD-induced extracellular matrix remodeling (Kelly et al., 2015). There are multiple differences between the two studies, including the species used, recording method, and selectivity for blocking MMP9 activity. However, a key difference may be that MMP9 knockout impairs potential developmental as well as ODP functions, while inactivation only during MD avoids this confound. Thus, MMP9 may indeed have a non-homeostatic role in open eye potentiation. Unexpectedly, astrocytic connexins were recently found to promote the maturation of inhibitory circuits and the extracellular matrix to close the critical period, and this process was suggested to occur through connexin mediated downregulation of MMP9 through the downregulation of RhoA activity (Ribot et al., 2021). It is unclear how astrocytic regulation of MMP9 and RhoA could influence heterosynaptic effects, or indeed relate to the role of astrocytic Ca^2+^ activity in heterosynaptic plasticity (Letellier et al., 2016).

Perhaps the most crucial role of astrocytes in regulating synaptic function is the clearance of extracellular glutamate *via* amino acid transporters. GLT1 is an astrocytic glutamate transporter responsible for 80–90% of synaptic glutamate clearance in the adult mouse cortex, and neuronal activity regulates the expression and subcellular trafficking of GLT1 to active synapses (Benediktsson et al., 2012). GLT1 expression in V1 begins at eye opening and peaks at the start of the critical period (Sipe et al., 2021). GLT1 heterozygous mice have a 40% reduction in GLT1 expression, and in V1 this reduction leads to mismatched contralateral and ipsilateral orientation selective responses in layer 2/3 neurons, with the ipsilateral responses abnormally high and poorly tuned relative to controls. These neurons also have increased synaptic density on their basal dendrites, which may indicate leakage of synaptic glutamate to neighboring spines triggering inadvertent H-LTP. Following MD, GLT1 heterozygous mice have normal deprived eye depression at 4 days, but a further reduction in both deprived eye and open eye (ipsilateral) responses at 7 days. Interestingly, GLT1 heterozygous mice, but not controls, have an increase in GLT1 expression at 4 and 7-days post MD. Therefore, the abnormal open eye depression may in part arise through the restoration of sufficient glutamate clearance to reduce unintentional synaptic crosstalk. As is becoming clear, astrocytes influence ODP through far more than the release of TNFα (Stellwagen and Malenka, 2006; Kaneko et al., 2008), and there are potentially many contributions that still remain unexplored (Muller and Best, 1989; Singh et al., 2016; Ribot et al., 2021).

Potentially downstream of NMDA receptor activation, CaMKII and CaMKII autophosphorylation are required for normal critical period ODP (Gordon et al., 1996; Taha and Stryker, 2002, 2005). Overexpression of calcineurin, on the other hand, prevents both ODP and the closure of the critical period (Yang et al., 2005). Thus, multiple sensors of intracellular Ca^2+^ are involved in the expression of ODP, but it remained unclear whether their activity was required postsynaptically. Using a FRET based sensor of active CaMKII, Mower et al. (2011) tracked synaptic CaMKII dynamics following brief MD in ferrets. Surprisingly, MD increased synaptic CaMKII activation in the deprived, but not open, eye domains. Providing perhaps the clearest demonstration of heterosynaptic mechanisms contributing to ODP, the spines eliminated in the deprived eye domains were those with low CaMKII activity while those with elevated CaMKII were protected. This suggests that elevated CaMKII in homosynaptically activated spines drives signaling that led to the depression of neighboring spines with low CaMKII activity, reminiscent of Arc targeting inactive synapses *via* binding to inactive CaMKIIβ (Okuno et al., 2012). Indeed, Arc is required for both deprived eye depression and open eye potentiation following critical period MD (McCurry et al., 2010), and overexpression of Arc is sufficient to restore critical period-like ODP to the adult V1 (Jenks et al., 2017). Thus, deprived eye depression may involve H-LTD of inactive dendritic spines through CaMKII and Arc. It is less clear why Arc would be required for open eye potentiation. It may be that, in the normal course of ODP, Arc-mediated endocytosis of AMPA receptors from deprived eye synapses forms a dendritic pool of available AMPA receptors for insertion into new or existing open eye synapses. Hinting at a conserved role for Arc in developmental plasticity brain-wide, Arc is also required for the elimination of excess climbing fiber to Purkinje cell synapses in the developing cerebellum (Mikuni et al., 2013), and for a critical period of spatial learning in the hippocampus (Gao et al., 2018).

While RhoA may contribute to the closure of the critical period through astrocytes (Ribot et al., 2021), upregulation of two other GTPases, H-Ras and Rac1, both mediators of LTP and H-LTP (Harvey et al., 2008; Hedrick et al., 2016), accelerate open eye potentiation in critical period and adult ODP respectively (Kaneko et al., 2010; Cerri et al., 2011). Inhibition of ERK suppresses open eye potentiation and cortical LTP (Di Cristo et al., 2001; Dumoulin et al., 2015), making a strong case for LTP or H-LTP mechanisms contributing to open eye potentiation. However, both RhoA and Rac1 regulated by NOGO-A have important roles in neurite outgrowth (Niederöst et al., 2002), which may drive open eye potentiation through increasing thalamocortical input to layer 4 (Cerri et al., 2011). There is significant potential to apply FRET based imaging approaches to examine synaptic GTPase activity following MD (Harvey et al., 2008; Murakoshi et al., 2011; Hedrick et al., 2016), and methods of restricting the spread of GTPase activity (Hedrick et al., 2016), to address to what degree both LTP and H-LTP contribute to open eye potentiation.

It should be noted that while we have largely focused on NMDA receptor dependent plasticity, in V1 the mechanisms of LTD are layer dependent with layer 4 LTD requiring NMDA receptor activity and AMPA receptor endocytosis, while layer 2/3 LTD requires endocannabinoid signaling and does not require AMPA receptor endocytosis (Crozier et al., 2007). Additionally, while mGluRs do not mediate layer 4 LTD or ODP (Hensch and Stryker, 1996; Sidorov et al., 2015), mGluR5 is necessary for the developmental NR2B to NR2A switch (Matta et al., 2011) and chronic mGluR suppression does indeed impair NMDA receptor dependent LTD and ODP (Dölen et al., 2007; Sidorov et al., 2015). Two other regulators of heterosynaptic plasticity, calcineurin, and β-Catenin, also regulate the function of NMDA receptors (Krupp et al., 2002; Saiepour et al., 2018; Tong et al., 2021). Through these feedback mechanisms, NMDA receptor function itself can be modulated in a synapse specific manner based on its prior history, allowing both heterosynaptic and metaplastic regulation of synaptic strength through NMDA receptors. In conclusion, many molecular mediators of heterosynaptic plasticity described *in vitro* are required for or interfere with ODP *in vivo*. Resolving to what extent their influence on ODP can be attributed to their heterosynaptic function is an exciting next step for future investigations.

## Role of Heterosynaptic Plasticity in Experience-Dependent Developmental Plasticity

We know a great deal about heterosynaptic plasticity and its molecular mechanisms, but we still know very little about the function of heterosynaptic plasticity *in vivo*. This discrepancy can be attributed to several causes. (1) Heterosynaptic plasticity is best observed in preparations with segregated input pathways or at the resolution of single synapses in combination with glutamate uncaging and/or functional indicators (Lynch et al., 1977; Callaway and Katz, 1993). In many brain regions, and particularly in the sensory cortices, inputs carry diverse, unsegregated information from the periphery, and glutamate uncaging and imaging at single spines *in vivo* remains technically challenging (Noguchi et al., 2011, 2019). (2) Most studies of heterosynaptic plasticity *in vitro* track changes induced by carefully controlled and calibrated stimuli over the course of minutes or hours. Developmental changes, on the other hand, occur over days or even weeks through naturalistic experience. Chronic structural imaging of dendritic spines during development has been feasible for many years (Majewska and Sur, 2003; Oray et al., 2004; Tropea et al., 2010), but structural evidence alone is insufficient to conclude heterosynaptic plasticity is required. (3) Finally, elucidating the molecular mediators of heterosynaptic plasticity has greatly benefited from targeted gene manipulation pre or post-synaptically, and the application of specialized indicators of protein activation (Harvey et al., 2008; Murakoshi et al., 2011; Hedrick et al., 2016). *In vivo* studies, on the other hand, have largely relied on whole animal or neuronal population knockout or overexpression of key genes, and many specialized sensors do not have the dynamic range required for imaging *in vivo* at a synaptic resolution. It is only through the iterative refinement of *in vivo* imaging techniques, functional indicators, and genetic tools for hypothesis-driven single-cell manipulation that the field has begun to make inroads into investigating the function of heterosynaptic plasticity *in vivo* (Mower et al., 2011; El-Boustani et al., 2018).

**Figure 5:**
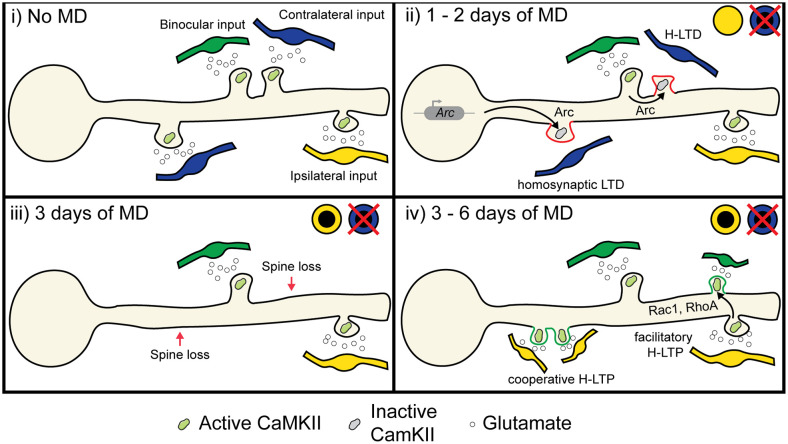
Proposed role of heterosynaptic plasticity in ODP. (i) During normal vision, monocular inputs from the contralateral and ipsilateral eye, as well as binocular inputs, converge onto neurons in the binocular visual cortex. Some visual inputs cluster for similar receptive field preferences and orientation preferences (Iacaruso et al., 2017; Lee et al., 2019). (ii) At 1 or 2 days of MD, low or unpatterned drive from deprived eye inputs lowers CaMKII activity in spines, which causes some spines to undergo homosynaptic LTD due to cell-wide transcription of Arc (McCurry et al., 2010), which binds with inactive CaMKII to induce AMPA receptor endocytosis (Okuno et al., 2012). Heterosynaptic LTD occurs in deprived eye inputs that are functionally clustered with open eye inputs due to the local translation and translocation of Arc (El-Boustani et al., 2018). (iii) At 3 days of MD, synapses exhibiting LTD are decreased in size or lost (Sun et al., 2019). (iv) After 6 days of MD, heterosynaptic potentiation occurs following spine loss (Frank et al., 2018). Existing open eye inputs facilitate the formation and strengthening of neighboring open eye synapses through the diffusion of activated GTPases such as Rac1 and RhoA (Hedrick et al., 2016). Furthermore, new clusters of open eye synapses also form through cooperative plasticity (Lee et al., 2016). Image assets reproduced from smart.servier.com (Servier Medical Art, 2015) (CC-BY).

Based on the molecular pathways shared between heterosynaptic plasticity and ODP (Table 1), we propose two mechanisms that could contribute to the depression of deprived eye inputs and potentiation of open eye inputs following MD. First, we hypothesize that both classical spike-timing-dependent homosynaptic LTD and compensatory H-LTD take place to weaken synapses from the deprived eye. Elevated CaMKII activation from open eye inputs could mediate heterosynaptic depression of neighboring inactive or weakly active deprived eye inputs by inducing the translation of dendritic *Arc* mRNA, leading to Arc protein binding to inactive CaMKIIβ and initiating AMPA receptor endocytosis (Figure 5 ii; Okuno et al., 2012; El-Boustani et al., 2018). Open eye inputs arrive ipsilaterally from the thalamus, but callosal inputs from the contralateral binocular V1 also provide excitatory open eye input (Restani et al., 2009). Callosal inputs play a role in suppressing responses to the deprived eye, and while this likely occurs through increased callosal excitation of inhibitory neurons, they may also provide excitatory drive onto dendritic spines that initiate H-LTD. Indeed, callosal and non-callosal inputs cluster for similar orientation preferences (Lee et al., 2019), therefore crosstalk between the two inputs is feasible and can explain why interocular alignment of orientation preference at the somatic level is disrupted during MD (Wang et al., 2010; Wang et al., 2013), as matched deprived eye inputs would be more likely to undergo H-LTD following MD than non-clustered inputs. This hypothesis predicts that elimination or weakening of deprived eye responsive synapses following MD is dependent on their distance from active, open eye responsive synapses.

The second mechanism we propose would occur during open eye response potentiation. As we have already discussed, feedback homeostatic synaptic scaling has been suggested as a potential mechanism for strengthening open eye inputs. However, the requirement of canonical LTP-mediating molecular factors, the potential absence of deprived eye potentiation (Gordon et al., 1996; Frenkel and Bear, 2004), and persistence of adult open eye potentiation with little deprived eye depression suggests that homeostasis alone does not fully explain the phenomena. We propose that open eye inputs are also strengthened through facilitatory and cooperative heterosynaptic potentiation. Such potentiation could be guided by the homeostatic strengthening of existing open eye inputs promoting the stabilization of new or neighboring open eye inputs. Indeed, prior activation of an existing spine increases the probability of subsequent glutamate release onto the adjacent dendritic shaft forming a new spine (Kwon and Sabatini, 2011), and the probability of new spine formation could be further increased on recent sites of synapse elimination following deprived eye depression (Figure 5 iii-iv; Frank et al., 2018). The shift in NMDA receptor composition following MD could also serve to lower the threshold for LTP (Chen and Bear, 2007; Cho et al., 2009), thus further decreasing the barrier for the formation or heterosynaptic strengthening of open eye synapses. A prediction of this hypothesis is that during late MD, open eye potentiation does not occur globally and multiplicatively across the neuron, but instead new or existing open eye inputs are more likely to strengthen if they are located near an existing open eye input. This H-LTP could be locally coordinated with earlier deprived eye synapse elimination, where H-LTD driven by an open eye synapse is followed by later H-LTP of new or previously weak open eye synapses. However, while the net loss of spines after 3 days MD is balanced by increased spine formation after 6 days MD (Sun et al., 2019), it remains unclear whether lost spines and newly formed spine are those responding to the closed eye and open eye respectively.

To determine whether heterosynaptic mechanisms contribute to the early and late phases of ODP, future work needs to be done *in vivo* at the synaptic level to distinguish deprived eye from open eye inputs on dendritic spines. By comparing structural plasticity between neighboring and distant synapses, and relating the plasticity to the visual activity of dendritic spines, it is possible to test if: (1) the initial depression of deprived eye inputs is more likely to occur when they are in close proximity to open eye inputs; and (2) open eye inputs are more likely to form or potentiate near existing open eye inputs and sites of recent synaptic loss. Furthermore, the possible molecular pathways that facilitate these heterosynaptic interactions can be screened in the same synapses imaged *in vivo* by FRET-based imaging or *post hoc* immunohistochemical staining for molecules involved in the induction and spread of heterosynaptic plasticity; including Arc, CaMKII, GTPases, NMDA receptor subunits, and AMPA receptor subunits. By combining the functional properties, structural plasticity, and molecular signatures of dendritic spines, we can establish a comprehensive picture of how neighboring synapses heterosynaptically interact within dendrites during ODP and other forms of developmental plasticity.

While this review has focused on the development of V1 and ODP in particular, it is equally important to examine the role of heterosynaptic plasticity in the development of other brain regions (Royer and Paré, 2003; Chu et al., 2015; Field et al., 2020; Mendes et al., 2020). While many functions of heterosynaptic plasticity in development are likely to be generalizable; from previous studies we expect plasticity rules to vary between different neuron types, brain regions, and developmental stages. In V1, heterosynaptic plasticity of non-stimulated inputs on fast-spiking interneurons functions to renormalize net input while in non-fast spiking interneurons heterosynaptic change is instead biased towards overall potentiation (Chistiakova et al., 2019). In the striatum, heterosynaptic plasticity rules differ between dopamine receptor 2 expressing and non-expressing medium spiny neurons (Mendes et al., 2020). In CA1, the spread of heterosynaptic plasticity through Ca^2+^ induced Ca^2+^ release declines over development (Lee et al., 2016), and in adult-born dentate granule cells, homosynaptic plasticity appears approximately 7 days prior to heterosynaptic plasticity and does not fully develop until much later (Jungenitz et al., 2018). It is not entirely clear how these neuron type, brain region, and developmental differences are expressed at the molecular level; however, changes in the expression or localization of many of the molecules discussed in this review are likely to be involved. Resolving to what extent the heterosynaptic function of these molecules is involved in aspects of developmental plasticity, such as ODP, is an exciting avenue through which we can begin to gain a fuller understanding of the experience-dependent development of neuronal circuits.

## Author Contributions

All authors contributed to the formulation of the novel concepts discussed in the review. KJ and KT wrote the review, with input and feedback from JZ, JI, and MS. All authors contributed to the article and approved the submitted version.

## Conflict of Interest

The authors declare that the research was conducted in the absence of any commercial or financial relationships that could be construed as a potential conflict of interest.

## Publisher’s Note

All claims expressed in this article are solely those of the authors and do not necessarily represent those of their affiliated organizations, or those of the publisher, the editors and the reviewers. Any product that may be evaluated in this article, or claim that may be made by its manufacturer, is not guaranteed or endorsed by the publisher.

## References

[B1] Anomal R. de Villers-Sidani E. Merzenich M. M. Panizzutti R. (2013). Manipulation of BDNF signaling modifies the experience-dependent plasticity induced by pure tone exposure during the critical period in the primary auditory cortex. PLoS One 8:e64208. 10.1371/journal.pone.0064208 23700463 PMC3660256

[B2] Baho E. Chattopadhyaya B. Lavertu-Jolin M. Mazziotti R. Awad P. N. Chehrazi P.. (2019). p75 neurotrophin receptor activation regulates the timing of the maturation of cortical parvalbumin interneuron connectivity and promotes juvenile-like plasticity in adult visual cortex. J. Neurosci. 39, 4489–4510. 10.1523/JNEUROSCI.2881-18.2019 30936240 PMC6554620

[B3] Bannon N. M. Chistiakova M. Chen J. Y. Bazhenov M. Volgushev M. (2017). Adenosine shifts plasticity regimes between associative and homeostatic by modulating heterosynaptic changes. J. Neurosci. 37, 1439–1452. 10.1523/JNEUROSCI.2984-16.2016 28028196 PMC5299565

[B4] Barnes S. J. Franzoni E. Jacobsen R. I. Erdelyi F. Szabo G. Clopath C.. (2017). Deprivation-induced homeostatic spine scaling *in vivo* is localized to dendritic branches that have undergone recent spine loss. Neuron 96, 871–882.e5. 10.1016/j.neuron.2017.09.052 29107520 PMC5697914

[B5] Bear M. F. Kleinschmidt A. Gu Q. Singer W. (1990). Disruption of experience-dependent synaptic modifications in striate cortex by infusion of an NMDA receptor antagonist. J. Neurosci 10, 909–925. 10.1523/JNEUROSCI.10-03-00909.1990 1969466 PMC6570130

[B6] Benediktsson A. M. Marrs G. S. Tu J. C. Worley P. F. Rothstein J. D. Bergles D. E.. (2012). Neuronal activity regulates glutamate transporter dynamics in developing astrocytes. Glia 60, 175–188. 10.1002/glia.21249 22052455 PMC3232333

[B7] Bian W. J. Miao W. Y. He S. J. Qiu Z. Yu X. (2015). Coordinated spine pruning and maturation mediated by inter-spine competition for cadherin/catenin complexes. Cell 162, 808–822. 10.1016/j.cell.2015.07.018 26255771

[B8] Bienenstock E. L. Cooper L. N. Munro P. W. (1982). Theory for the development of neuron selectivity: orientation specificity and binocular interaction in visual cortex. J. Neurosci. 2, 32–48. 10.1523/JNEUROSCI.02-01-00032.1982 7054394 PMC6564292

[B9] Bliss T. V. P. Collingridge G. L. (1993). A synaptic model of memory: LTP in the hippocampus. Nature 361, 31–39. 10.1038/361031a0 8421494

[B10] Bonhoeffer T. Staiger V. Aertsen A. (1989). Synaptic plasticity in rat hippocampal slice cultures: local “Hebbian” conjunction of pre- and postsynaptic stimulation leads to distributed synaptic enhancement. Proc. Natl. Acad. Sci. U S A 86, 8113–8117. 10.1073/pnas.86.20.8113 2813381 PMC298225

[B12] Cabelli R. Hohn A. Shatz C. (1995). Inhibition of ocular dominance column formation by infusion of NT-4/5 or BDNF. Science 267, 1662–1666. 10.1126/science.7886458 7886458

[B11] Cabelli R. J. Shelton D. L. Segal R. A. Shatz C. J. (1997). Blockade of endogenous ligands of TrkB inhibits formation of ocular dominance columns. Neuron 19, 63–76. 10.1016/s0896-6273(00)80348-7 9247264

[B13] Callaway E. M. Katz L. C. (1993). Photostimulation using caged glutamate reveals functional circuitry in living brain slices. Proc. Natl. Acad. Sci. U S A 90, 7661–7665. 10.1073/pnas.90.16.7661 7689225 PMC47202

[B500] Cao Z. Liu L. Lickey M. Graves A. Pham T. Gordon B. (2007). Virally mediated knock-down of NR2 subunits ipsilateral to the deprived eye blocks ocular dominance plasticity. Exp. Brain Res. 177, 64–77. 10.1007/s00221-006-0647-8 16944113

[B14] Carlson K. D. Richert M. Dutt N. Krichmar J. L. (2013). “Biologically plausible models of homeostasis and STDP: stability and learning in spiking neural networks,” The 2013 International Joint Conference on Neural Networks (IJCNN), 2013, 1–8. 10.1109/IJCNN.2013.6706961

[B15] Cash S. Yuste R. (1998). Input summation by cultured pyramidal neurons is linear and position-independent. J. Neurosci. 18, 10–15. 10.1523/JNEUROSCI.18-01-00010.1998 9412481 PMC6793421

[B16] Cash S. Yuste R. (1999). Linear summation of excitatory inputs by CA1 pyramidal neurons. Neuron 22, 383–394. 10.1016/s0896-6273(00)81098-3 10069343

[B17] Castro J. Garcia R. I. Kwok S. Banerjee A. Petravicz J. Woodson J.. (2014). Functional recovery with recombinant human IGF1 treatment in a mouse model of rett syndrome. Proc. Natl. Acad. Sci. U S A 111, 9941–9946. 10.1073/pnas.1311685111 24958891 PMC4103342

[B18] Cerri C. Fabbri A. Vannini E. Spolidoro M. Costa M. Maffei L.. (2011). Activation of Rho GTPases triggers structural remodeling and functional plasticity in the adult rat visual cortex. J. Neurosci. 31, 15163–15172. 10.1523/JNEUROSCI.2617-11.2011 22016550 PMC6623549

[B22] Chen W. S. Bear M. F. (2007). Activity-dependent regulation of NR2B translation contributes to metaplasticity in mouse visual cortex. Neuropharmacology 52, 200–214. 10.1016/j.neuropharm.2006.07.003 16895734

[B23] Chen X. Leischner U. Rochefort N. L. Nelken I. Konnerth A. (2011). Functional mapping of single spines in cortical neurons *in vivo*. Nature 475, 501–505. 10.1038/nature10193 21706031

[B20] Chen J. Y. Lonjers P. Lee C. Chistiakova M. Volgushev M. Bazhenov M. (2013). Heterosynaptic plasticity prevents runaway synaptic dynamics. J. Neurosci. 33, 15915–15929. 10.1523/JNEUROSCI.5088-12.2013 24089497 PMC3787503

[B19] Chen J. L. Villa K. L. Cha J. W. So P. T. C. Kubota Y. Nedivi E. (2012). Clustered dynamics of inhibitory synapses and dendritic spines in the adult neocortex. Neuron 74, 361–373. 10.1016/j.neuron.2012.02.030 22542188 PMC3340582

[B21] Chen T.-W. Wardill T. J. Sun Y. Pulver S. R. Renninger S. L. Baohan A.. (2013). Ultrasensitive fluorescent proteins for imaging neuronal activity. Nature 499, 295–300. 10.1038/nature12354 23868258 PMC3777791

[B24] Chistiakova M. Ilin V. Roshchin M. Bannon N. Malyshev X. A. Kisvárday X. Z.. (2019). Distinct heterosynaptic plasticity in fast spiking and non-fast-spiking inhibitory neurons in rat visual cortex. J. Neurosci. 39, 6865–6878. 10.1523/JNEUROSCI.3039-18.2019 31300522 PMC6733570

[B25] Cho K. K. A. Khibnik L. Philpot B. D. Bear M. F. (2009). The ratio of NR2A/B NMDA receptor subunits determines the qualities of ocular dominance plasticity in visual cortex. Proc. Natl. Acad. Sci. U S A 106, 5377–5382. 10.1073/pnas.0808104106 19276107 PMC2654025

[B26] Chowdhury S. Shepherd J. D. Okuno H. Lyford G. Petralia R. S. Plath N.. (2006). Arc/Arg3.1 interacts with the endocytic machinery to regulate AMPA receptor trafficking. Neuron 52, 445–459. 10.1016/j.neuron.2006.08.033 17088211 PMC1784006

[B27] Chu H. Y. Atherton J. F. Wokosin D. Surmeier D. J. Bevan M. D. (2015). Heterosynaptic regulation of external globus pallidus inputs to the subthalamic nucleus by the motor cortex. Neuron 85, 364–376. 10.1016/j.neuron.2014.12.022 25578364 PMC4304914

[B28] Collingridge G. L. Kehl S. J. McLennan H. (1983). Excitatory amino acids in synaptic transmission in the schaffer collateral-commissural pathway of the rat hippocampus. J. Physiol. 334, 33–46. 10.1113/jphysiol.1983.sp014478 6306230 PMC1197298

[B29] Crozier R. A. Wang Y. Liu C. H. Bear M. F. (2007). Deprivation-induced synaptic depression by distinct mechanisms in different layers of mouse visual cortex. Proc. Natl. Acad. Sci. U S A 104, 1383–1388. 10.1073/pnas.0609596104 17227847 PMC1783104

[B30] Dakin S. Frith U. (2005). Vagaries of visual perception in autism. Neuron 48, 497–507. 10.1016/j.neuron.2005.10.018 16269366

[B31] Dallérac G. Zerwas M. Novikova T. Callu D. Leblanc-Veyrac P. Bock E.. (2011). The neural cell adhesion molecule-derived peptide FGL facilitates long-term plasticity in the dentate gyrus *in vivo*. Learn. Mem. 18, 306–313. 10.1101/lm.2154311 21508096

[B32] De Roo M. Klauser P. Muller D. (2008). LTP promotes a selective long-term stabilization and clustering of dendritic spines. PLoS Biol. 6:e219. 10.1371/journal.pbio.0060219 18788894 PMC2531136

[B33] Denk W. (1994). Two-photon scanning photochemical microscopy: mapping ligand-gated ion channel distributions. Proc. Natl. Acad. Sci. U S A 91, 6629–6633. 10.1073/pnas.91.14.6629 7517555 PMC44256

[B34] Di Cristo G. Berardi N. Cancedda L. Pizzorusso T. Putignano E. Ratto G. M.. (2001). Requirement of ERK activation for visual cortical plasticity. Science 292, 2337–2340. 10.1126/science.1059075 11423664

[B35] Dölen G. Osterweil E. Rao B. S. S. Smith G. B. Auerbach B. D. Chattarji S.. (2007). Correction of fragile X syndrome in mice. Neuron 56, 955–962. 10.1016/j.neuron.2007.12.001 18093519 PMC2199268

[B36] Dumoulin M. C. Aton S. J. Watson A. J. Renouard L. Coleman T. Frank M. G. (2015). Extracellular signal-regulated kinase (ERK) activity during sleep consolidates cortical plasticity *in vivo*. Cereb. Cortex 25, 507–515. 10.1093/cercor/bht250 24047601 PMC4303804

[B37] El-Boustani S. K Ip J. P. Breton-Provencher V. Knott G. W. Okuno H. Bito H.. (2018). Locally coordinated synaptic plasticity of visual cortex neurons *in vivo*. Science 360, 1340–1354. 10.1126/science.aao0862 29930137 PMC6366621

[B38] Engert F. Bonhoeffer T. (1997). Synapse specificity of long-term potentiation breaks down at short distances. Nature 388, 279–284. 10.1038/40870 9230437

[B39] Espinosa J. S. Stryker M. P. (2012). Development and plasticity of the primary visual cortex. Neuron 75, 230–249. 10.1016/j.neuron.2012.06.009 22841309 PMC3612584

[B40] Faust T. E. Gunner G. Schafer D. P. (2021). Mechanisms governing activity-dependent synaptic pruning in the developing mammalian CNS. Nat. Rev. Neurosci. 22, 657–673. 10.1038/s41583-021-00507-y 34545240 PMC8541743

[B41] Field R. E. D’amour J. A. Tremblay R. Miehl C. Rudy B. Gjorgjieva J.. (2020). Heterosynaptic plasticity determines the set point for cortical excitatory-inhibitory balance. Neuron 106, 842–854.e4. 10.1016/j.neuron.2020.03.002 32213321 PMC7274908

[B42] Fox K. Stryker M. (2017). Integrating hebbian and homeostatic plasticity: introduction. Philos. Trans. R Soc. Lond B Biol. Sci. 372:20160413. 10.1098/rstb.2016.0413 28093560 PMC5247598

[B43] Frank A. C. Huang S. Zhou M. Gdalyahu A. Kastellakis G. Silva T. K.. (2018). Hotspots of dendritic spine turnover facilitate clustered spine addition and learning and memory. Nat. Commun. 9:422. 10.1038/s41467-017-02751-2 29379017 PMC5789055

[B44] Frenkel M. Y. Bear M. F. (2004). How monocular deprivation shifts ocular dominance in visual cortex of young mice. Neuron 44, 917–923. 10.1016/j.neuron.2004.12.003 15603735

[B45] Frey U. Morris R. G. (1997). Synaptic tagging and LTP. Nature 385, 533–536. 10.1038/385533a0 9020359

[B46] Fu M. Yu X. Lu J. Zuo Y. (2012). Repetitive motor learning induces coordinated formation of clustered dendritic spines *in vivo*. Nature 483, 92–95. 10.1038/nature10844 22343892 PMC3292711

[B47] Galuske R. A. W. Kim D. Castreny E. Thoenen H. Singer W. (1996). Brain-derived neurotrophic factor reverses experience- dependent synaptic modifications in kitten visual cortex. Eur. J. Neurosci. 8, 1554–1559. 10.1111/j.1460-9568.1996.tb01618.x 8758963

[B48] Gao X. Castro-Gomez S. Grendel J. Graf S. Süsens U. Binkle L.. (2018). Arc/Arg3.1 mediates a critical period for spatial learning and hippocampal networks. Proc. Natl. Acad. Sci. U S A 115, 12531–12536. 10.1073/pnas.1810125115 30442670 PMC6298089

[B49] Gasparini S. Magee J. C. (2006). State-dependent dendritic computation in hippocampal CA1 pyramidal neurons. J. Neurosci. 26, 2088–2100. 10.1523/JNEUROSCI.4428-05.2006 16481442 PMC6674927

[B50] Gökçe O. Bonhoeffer T. Scheuss V. (2016). Clusters of synaptic inputs on dendrites of layer 5 pyramidal cells in mouse visual cortex. eLife 5:e09222. 10.7554/eLife.09222 27431612 PMC4951190

[B51] Gordon J. A. Cioffi D. Silva A. J. Stryker M. P. (1996). Deficient plasticity in the primary visual cortex of α- calcium/calmodulin-dependent protein kinase II mutant mice. Neuron 17, 491–499. 10.1016/s0896-6273(00)80181-6 8816712

[B52] Gordon U. Polsky A. Schiller J. (2006). Plasticity compartments in basal dendrites of neocortical pyramidal neurons. J. Neurosci. 26, 12717–12726. 10.1523/JNEUROSCI.3502-06.2006 17151275 PMC6674852

[B53] Govindarajan A. Israely I. Huang S. Y. Tonegawa S. (2011). The dendritic branch is the preferred integrative unit for protein synthesis-dependent LTP. Neuron 69, 132–146. 10.1016/j.neuron.2010.12.008 21220104 PMC3032443

[B54] Harnett M. T. Makara J. K. Spruston N. Kath W. L. Magee J. C. (2012). Synaptic amplification by dendritic spines enhances input cooperativity. Nature 491, 599–602. 10.1038/nature11554 23103868 PMC3504647

[B55] Harvey C. D. Svoboda K. (2007). Locally dynamic synaptic learning rules in pyramidal neuron dendrites. Nature 450, 1195–1200. 10.1038/nature06416 18097401 PMC3425382

[B56] Harvey C. D. Yasuda R. Zhong H. Svoboda K. (2008). The spread of ras activity triggered by activation of a single dendritic spine. Science 321, 133–136. 10.1126/science.1159675 18556515 PMC2745709

[B57] Hasselmo M. E. (1994). Runaway synaptic modification in models of cortex: implications for Alzheimer’s disease. Neural Netw. 7, 13–40. 10.1016/0893-6080(94)90053-1

[B58] Hedrick N. G. Harward S. C. Hall C. E. Murakoshi H. McNamara J. O. Yasuda R. (2016). Rho GTPase complementation underlies BDNF-dependent homo- and heterosynaptic plasticity. Nature 538, 104–108. 10.1038/nature19784 27680697 PMC5361895

[B59] Hensch T. K. (2005). Critical period plasticity in local cortical circuits. Nat. Rev. Neurosci. 6, 877–888. 10.1038/nrn1787 16261181

[B60] Hensch T. K. Stryker M. P. (1996). Ocular dominance plasticity under metabotropic glutamate receptor blockade. Science 272, 554–557. 10.1126/science.272.5261.554 8614806

[B61] Heynen A. J. Yoon B. Liu C. Chung H. J. Huganir R. L. Bear M. F. (2003). Molecular mechanism for loss of visual cortical responsiveness following brief monocular deprivation. Nat. Neurosci. 6, 854–862. 10.1038/nn1100 12886226

[B63] Hofer S. B. Mrsic-Flogel T. D. Bonhoeffer T. Hübener M. (2009). Experience leaves a lasting structural trace in cortical circuits. Nature 457, 313–317. 10.1038/nature07487 19005470 PMC6485433

[B64] Hoffman D. A. Magee J. C. Colbert C. M. Johnston D. (1997). K+ channel regulation of signal propagation in dendrites of hippocampal pyramidal neurons. Nature 387, 869–875. 10.1038/43119 9202119

[B65] Holtmaat A. J. G. D. Trachtenberg J. T. Wilbrecht L. Shepherd G. M. Zhang X. Knott G. W.. (2005). Transient and persistent dendritic spines in the neocortex *in vivo*. Neuron 45, 279–291. 10.1016/j.neuron.2005.01.003 15664179

[B66] Hooks B. M. Chen C. (2020). Circuitry underlying experience-dependent plasticity in the mouse visual system. Neuron 106, 21–36. 10.1016/j.neuron.2020.01.031 32272065 PMC7251959

[B67] Huang Z. J. Kirkwood A. Pizzorusso T. Porciatti V. Morales B. Bear M. F.. (1999). BDNF regulates the maturation of inhibition and the critical period of plasticity in mouse visual cortex. Cell 98, 739–755. 10.1016/s0092-8674(00)81509-3 10499792

[B68] Hubel D. H. Wiesel T. N. (1959). Receptive fields of single neurons in the cat’s striate cortex. J. Physiol. 148, 574–591. 10.1113/jphysiol.1959.sp006308 14403679 PMC1363130

[B69] Iacaruso M. F. Gasler I. T. Hofer S. B. (2017). Synaptic organization of visual space in primary visual cortex. Nature 547, 449–452. 10.1038/nature23019 28700575 PMC5533220

[B70] Ip J. P. K. Nagakura I. Petravicz J. Li K. Wiemer E. A. C. Sur M. (2018). Major vault protein, a candidate gene in 16p11.2 microdeletion syndrome, is required for the homeostatic regulation of visual cortical plasticity. J. Neurosci. 38, 3890–3900. 10.1523/JNEUROSCI.2034-17.2018 29540554 PMC5907052

[B71] Jakkamsetti V. Tsai N. Gross C. Molinaro G. Collins K. A. Nicoletti F.. (2013). Experience-induced Arc/Arg3.1 primes CA1 pyramidal neurons for metabotropic glutamate receptor-dependent long-term synaptic depression. Neuron 80, 72–79. 10.1016/j.neuron.2013.07.020 24094104 PMC3801421

[B72] Jenks K. R. Kim T. Pastuzyn E. D. Okuno H. Taibi A. V. Bito H.. (2017). Arc restores juvenile plasticity in adult mouse visual cortex. Proc. Natl. Acad. Sci. U S A 114, 9182–9187. 10.1073/pnas.1700866114 28790183 PMC5576785

[B74] Jia H. Varga Z. Sakmann B. Konnerth A. (2014). Linear integration of spine Ca2+ signals in layer 4 cortical neurons *in vivo*. Proc. Natl. Acad. Sci. U S A 111, 9277–9282. 10.1073/pnas.1408525111 24927564 PMC4078833

[B75] Johnston D. Magee J. C. Colbert C. M. Christie B. R. (1996). Active properties of neuronal dendrites. Annu. Rev. Neurosci. 19, 165–186. 10.1146/annurev.ne.19.030196.001121 8833440

[B76] Ju N. Li Y. Liu F. Jiang H. Macknik S. L. Martinez-Conde S.. (2020). Spatiotemporal functional organization of excitatory synaptic inputs onto macaque V1 neurons. Nat. Commun. 11:697. 10.1038/s41467-020-14501-y 32019929 PMC7000673

[B77] Jungenitz T. Beining M. Radic T. Deller T. Cuntz H. Jedlicka P.. (2018). Structural homo- and heterosynaptic plasticity in mature and adult newborn rat hippocampal granule cells. Proc. Natl. Acad. Sci. U S A 115, E4670–E4679. 10.1073/pnas.1801889115 29712871 PMC5960324

[B78] Kaneko M. Cheetham C. E. Lee Y. S. Silva A. J. Stryker M. P. Fox K. (2010). Constitutively active H-ras accelerates multiple forms of plasticity in developing visual cortex. Proc. Natl. Acad. Sci. U S A 107, 19026–19031. 10.1073/pnas.1013866107 20937865 PMC2973899

[B79] Kaneko M. Stellwagen D. Malenka R. C. Stryker M. P. (2008). Tumor necrosis factor-α mediates one component of competitive, experience-dependent plasticity in developing visual cortex. Neuron 58, 673–680. 10.1016/j.neuron.2008.04.023 18549780 PMC2884387

[B80] Kelly E. A. Russo A. S. Jackson C. D. Lamantia C. E. Ania K. (2015). Proteolytic regulation of synaptic plasticity in the mouse primary visual cortex: analysis of matrix metalloproteinase 9 deficient mice. Front. Cell. Neurosci. 9:369. 10.3389/fncel.2015.00369 26441540 PMC4585116

[B81] Kerlin A. Mohar B. Flickinger D. Maclennan B. J. Dean M. B. Davis C.. (2019). Functional clustering of dendritic activity during decision-making. eLife 8:e46966. 10.7554/eLife.46966 31663507 PMC6821494

[B82] Kirchner J. H. Gjorgjieva J. (2021). Emergence of local and global synaptic organization on cortical dendrites. Nat. Commun. 12:4005. 10.1038/s41467-021-23557-3 34183661 PMC8239006

[B83] Kleindienst T. Winnubst J. Roth-Alpermann C. Bonhoeffer T. Lohmann C. (2011). Activity-dependent clustering of functional synaptic inputs on developing hippocampal dendrites. Neuron 72, 1012–1024. 10.1016/j.neuron.2011.10.015 22196336

[B85] Kossel A. Bonhoeffer T. Bolz J. (1990). Non-Hebbian synapses in rat visual cortex. Neuroreport 1, 115–118. 10.1097/00001756-199010000-00008 2129865

[B86] Krupp J. J. Vissel B. Thomas C. G. Heinemann S. F. Westbrook G. L. (2002). Calcineurin acts *via* the C-terminus of NR2A to modulate desensitization of NMDA receptors. Neuropharmacology 42, 593–602. 10.1016/s0028-3908(02)00031-x 11985816

[B87] Kumar S. Kumar M. P. Kaushik Y. Jayaprakash B. (2020). Clustered loss of dendritic spines characterizes encoding of related memory. bioRxiv [Preprint]. 10.1101/2020.12.17.423264

[B88] Kwon H. B. Sabatini B. L. (2011). Glutamate induces de novo growth of functional spines in developing cortex. Nature 474, 100–104. 10.1038/nature09986 21552280 PMC3107907

[B89] Lai C. S. W. Franke T. F. Gan W. B. (2012). Opposite effects of fear conditioning and extinction on dendritic spine remodelling. Nature 483, 87–91. 10.1038/nature10792 22343895

[B90] Lambo M. E. Turrigiano G. G. (2013). Synaptic and intrinsic homeostatic mechanisms cooperate to increase L2/3 pyramidal neuron excitability during a late phase of critical period plasticity. J. Neurosci. 33, 8810–8819. 10.1523/JNEUROSCI.4502-12.2013 23678123 PMC3700430

[B91] Leblanc J. J. Fagiolini M. (2011). Autism: a critical period disorder? Neural Plast. 2011:921680. 10.1155/2011/921680 21826280 PMC3150222

[B92] Lee K. F. H. Soares C. Thivierge J. P. Béïque J. C. (2016). Correlated synaptic inputs drive dendritic calcium amplification and cooperative plasticity during clustered synapse development. Neuron 89, 784–799. 10.1016/j.neuron.2016.01.012 26853305

[B93] Lee K. S. Vandemark K. Mezey D. Shultz N. Fitzpatrick D. (2019). Functional synaptic architecture of callosal inputs in mouse primary visual cortex. Neuron 101, 421–428.e5. 10.1016/j.neuron.2018.12.005 30658859 PMC7012385

[B94] Legenstein R. Maass W. (2011). Branch-specific plasticity enables self-organization of nonlinear computation in single neurons. J. Neurosci. 31, 10787–10802. 10.1523/JNEUROSCI.5684-10.2011 21795531 PMC6623094

[B95] Letellier M. Levet F. Thoumine O. Goda Y. (2019). Differential role of pre-and postsynaptic neurons in the activity-dependent control of synaptic strengths across dendrites. PLoS Biol. 17:e2006223. 10.1371/journal.pbio.2006223 31166943 PMC6576792

[B96] Letellier M. Park Y. K. Chater T. E. Chipman P. H. Gautam S. G. Oshima-Takago T.. (2016). Astrocytes regulate heterogeneity of presynaptic strengths in hippocampal networks. Proc. Natl. Acad. Sci. U S A 113, E2685–E2694. 10.1073/pnas.1523717113 27118849 PMC4868440

[B97] Levelt C. N. Hübener M. (2012). Critical-period plasticity in the visual cortex. Annu. Rev. Neurosci. 35, 309–330. 10.1146/annurev-neuro-061010-113813 22462544

[B98] Limbacher T. Legenstein R. (2020). Emergence of stable synaptic clusters on dendrites through synaptic rewiring. Front. Comput. Neurosci. 14:57. 10.3389/fncom.2020.00057 32848681 PMC7424032

[B99] Losonczy A. Magee J. C. (2006). Integrative properties of radial oblique dendrites in hippocampal CA1 pyramidal neurons. Neuron 50, 291–307. 10.1016/j.neuron.2006.03.016 16630839

[B100] Lyford G. L. Yamagata K. Kaufmann W. E. Barnes C. A. Sanders L. K. Copeland N. G.. (1995). Arc, a growth factor and activity-regulated gene, encodes a novel cytoskeleton-associated protein that is enriched in neuronal dendrites. Neuron 14, 433–445. 10.1016/0896-6273(95)90299-6 7857651

[B101] Lynch G. S. Dunwiddie T. Gribkoff V. (1977). Heterosynaptic depression: a postsynaptic correlate of long-term potentiation. Nature 266, 737–739. 10.1038/266737a0 195211

[B102] Magó Á. Weber J. P. Ujfalussy B. B. Makara J. K. (2020). Synaptic plasticity depends on the fine-scale input pattern in thin dendrites of CA1 pyramidal neurons. J. Neurosci. 40, 2593–2605. 10.1523/JNEUROSCI.2071-19.2020 32047054 PMC7096145

[B103] Majewska A. Sur M. (2003). Motility of dendritic spines in visual cortex *in vivo*: changes during the critical period and effects of visual deprivation. Proc. Natl. Acad. Sci. U S A 100, 16024–16029. 10.1073/pnas.2636949100 14663137 PMC307686

[B104] Makino H. Malinow R. (2011). Compartmentalized versus global synaptic plasticity on dendrites controlled by experience. Neuron 72, 1001–1011. 10.1016/j.neuron.2011.09.036 22196335 PMC3310180

[B105] Malenka R. C. Bear M. F. (2004). LTP and LTD: an embarrassment of riches. Neuron 44, 5–21. 10.1016/j.neuron.2004.09.012 15450156

[B106] Matta J. A. Ashby M. C. Sanz-Clemente A. Roche K. W. Isaac J. T. R. (2011). MGluR5 and NMDA receptors drive the experience- and activity-dependent NMDA receptor NR2B to NR2A subunit switch. Neuron 70, 339–351. 10.1016/j.neuron.2011.02.045 21521618 PMC3087383

[B107] McCurry C. L. Shepherd J. D. Tropea D. Wang K. H. Bear M. F. Sur M. (2010). Loss of Arc renders the visual cortex impervious to the effects of sensory experience or deprivation. Nat. Neurosci. 13, 450–457. 10.1038/nn.2508 20228806 PMC2864583

[B108] Mel B. W. (1993). Synaptic integration in an excitable dendritic tree. J. Neurophysiol. 70, 1086–1101. 10.1152/jn.1993.70.3.1086 8229160

[B109] Mendes A. Vignoud G. Perez S. Perrin E. Touboul J. Venance L. (2020). Concurrent thalamostriatal and corticostriatal spike-timing-dependent plasticity and heterosynaptic interactions shape striatal plasticity map. Cereb. Cortex 30, 4381–4401. 10.1093/cercor/bhaa024 32147733

[B110] Meredith R. M. (2015). Sensitive and critical periods during neurotypical and aberrant neurodevelopment: a framework for neurodevelopmental disorders. Neurosci. Biobehav. Rev. 50, 180–188. 10.1016/j.neubiorev.2014.12.001 25496903

[B111] Mikuni T. Uesaka N. Okuno H. Hirai H. Deisseroth K. Bito H.. (2013). Arc/Arg3.1 is a postsynaptic mediator of activity-dependent synapse elimination in the developing cerebellum. Neuron 78, 1024–1035. 10.1016/j.neuron.2013.04.036 23791196 PMC3773328

[B112] Miquelajauregui A. Kribakaran S. Mostany R. Badaloni A. Consalez G. G. Portera-Cailliau C. (2015). Layer 4 pyramidal neurons exhibit robust dendritic spine plasticity *in vivo* after input deprivation. J. Neurosci. 35, 7287–7294. 10.1523/JNEUROSCI.5215-14.2015 25948276 PMC4420789

[B113] Mower A. F. Kwok S. Yu H. Majewska A. K. Okamoto K. I. Hayashi Y.. (2011). Experience-dependent regulation of CaMKII activity within single visual cortex synapses *in vivo*. Proc. Natl. Acad. Sci. U S A 108, 21241–21246. 10.1073/pnas.1108261109 22160721 PMC3248554

[B114] Mrsic-Flogel T. D. Hofer S. B. Ohki K. Reid R. C. Bonhoeffer T. Hübener M. (2007). Homeostatic regulation of eye-specific responses in visual cortex during ocular dominance plasticity. Neuron 54, 961–972. 10.1016/j.neuron.2007.05.028 17582335

[B115] Muller C. M. Best J. (1989). Ocular dominance plasticity in adult cat visual cortex after transplantation of cultured astrocytes. Nature 342, 427–430. 10.1038/342427a0 2586611

[B116] Mullins C. Fishell G. Tsien R. W. (2016). Unifying views of autism spectrum disorders: a consideration of autoregulatory feedback loops. Neuron 89, 1131–1156. 10.1016/j.neuron.2016.02.017 26985722 PMC5757244

[B117] Murakoshi H. Wang H. Yasuda R. (2011). Local, persistent activation of Rho GTPases during plasticity of single dendritic spines. Nature 472, 100–104. 10.1038/nature09823 21423166 PMC3105377

[B118] Murase S. Winkowski D. E. Liu J. Kanold P. O. Quinlan E. M. (2019). Homeostatic regulation of perisynaptic matrix metalloproteinase 9 (MMP9) activity in the amblyopic visual cortex. eLife 8:e52503. 10.7554/eLife.52503 31868167 PMC6961978

[B119] Nagakura I. Van Wart A. Petravicz J. Tropea D. Sur M. (2014). STAT1 regulates the homeostatic component of visual cortical plasticity *via* an AMPA receptor-mediated mechanism. J. Neurosci. 34, 10256–10263. 10.1523/JNEUROSCI.0189-14.2014 25080587 PMC4115137

[B120] Nelson S. B. Valakh V. (2015). Excitatory/inhibitory balance and circuit homeostasis in autism spectrum disorders. Neuron 87, 684–698. 10.1016/j.neuron.2015.07.033 26291155 PMC4567857

[B121] Niculescu D. Michaelsen-Preusse K. Güner Ü. van Dorland R. Wierenga C. J. Lohmann C. (2018). A BDNF-mediated push-pull plasticity mechanism for synaptic clustering. Cell Rep. 24, 2063–2074. 10.1016/j.celrep.2018.07.073 30134168

[B122] Niederöst B. Oertle T. Fritsche J. McKinney R. A. Bandtlow C. E. (2002). Nogo-A and myelin-associated glycoprotein mediate neurite growth inhibition by antagonistic regulation of RhoA and Rac1. J. Neurosci. 22, 10368–10376. 10.1016/j.isci.2021.103301 12451136 PMC6758757

[B123] Nishiyama M. Hong K. Mikoshiba K. Poo M. M. Kato K. (2000). Calcium stores regulate the polarity and input specificity of synaptic modification. Nature 408, 584–588. 10.1038/35046067 11117745

[B124] Noguchi J. Nagaoka A. Hayama T. Ucar H. Yagishita S. Takahashi N.. (2019). Bidirectional *in vivo* structural dendritic spine plasticity revealed by two-photon glutamate uncaging in the mouse neocortex. Sci. Rep. 9:13922. 10.1038/s41598-019-50445-0 31558759 PMC6763442

[B125] Noguchi J. Nagaoka A. Watanabe S. Ellis-Davies G. C. R. Kitamura K. Kano M.. (2011). *in vivo* two-photon uncaging of glutamate revealing the structure-function relationships of dendritic spines in the neocortex of adult mice. J. Physiol. 589, 2447–2457. 10.1113/jphysiol.2011.207100 21486811 PMC3115818

[B126] Oh W. C. Parajuli L. K. Zito K. (2015). Heterosynaptic structural plasticity on local dendritic segments of hippocampal CA1 neurons. Cell Rep. 10, 162–169. 10.1016/j.celrep.2014.12.016 25558061 PMC4294981

[B127] Okuno H. Akashi K. Ishii Y. Yagishita-Kyo N. Suzuki K. Nonaka M.. (2012). Inverse synaptic tagging of inactive synapses *via* dynamic interaction of Arc/Arg3.1 with CaMKIIβ. Cell 149, 886–898. 10.1016/j.cell.2012.02.062 22579289 PMC4856149

[B128] Oray S. Majewska A. Sur M. (2004). Dendritic spine dynamics are regulated by monocular deprivation and extracellular matrix degradation. Neuron 44, 1021–1030. 10.1016/j.neuron.2004.12.001 15603744

[B130] Park S. Park J. M. Kim S. Kim J.-A. Shepherd J. D. Smith-Hicks C. L.. (2008). Elongation factor 2 and fragile X mental retardation protein control the dynamic translation of Arc/Arg3.1 essential for mGluR-LTD. Neuron 59, 70–83. 10.1016/j.neuron.2008.05.023 18614030 PMC2743934

[B131] Pastuzyn E. D. Day C. E. Kearns R. B. Kyrke-Smith M. Taibi A. V. McCormick J.. (2018). The neuronal gene arc encodes a repurposed retrotransposon gag protein that mediates intercellular RNA transfer. Cell 172, 275–288.e18. 10.1016/j.cell.2017.12.024 29328916 PMC5884693

[B132] Pizzorusso T. Medini P. Nicoletta B. Chierzi S. Fawcett J. Maffei L. (2002). Reactivation of ocular dominance plasticity in the adult visual cortex. Science 298, 1248–1251. 10.1126/science.1072699 12424383

[B133] Plath N. Ohana O. Dammermann B. Errington M. L. Schmitz D. Gross C.. (2006). Arc/Arg3.1 is essential for the consolidation of synaptic plasticity and memories. Neuron 52, 437–444. 10.1016/j.neuron.2006.08.024 17088210

[B134] Rall W. (1967). Distinguishing theoretical synaptic poten- tials computed for different soma-den- dritic distributions of synaptic. J. Physiol. 30, 1138–1168. 10.1152/jn.1967.30.5.1138 6055351

[B135] Ranson A. Cheetham C. E. J. Fox K. Sengpiel F. (2012). Homeostatic plasticity mechanisms are required for juvenile, but not adult, ocular dominance plasticity. Proc. Natl. Acad. Sci. U S A 109, 1311–1316. 10.1073/pnas.1112204109 22232689 PMC3268335

[B136] Restani L. Cerri C. Pietrasanta M. Gianfranceschi L. Maffei L. Caleo M. (2009). Functional masking of deprived eye responses by callosal input during ocular dominance plasticity. Neuron 64, 707–718. 10.1016/j.neuron.2009.10.019 20005826

[B137] Ribot J. Breton R. Calvo C. F. Moulard J. Ezan P. Zapata J.. (2021). Astrocytes close the mouse critical period for visual plasticity. Science 373, 77–81. 10.1126/science.abf5273 34210880

[B138] Roberts E. B. Meredith M. A. Ramoa A. S. (1998). Suppression of NMDA receptor function using antisense DNA block ocular dominance plasticity while preserving visual responses. J. Neurophysiol. 80, 1021–1032. 10.1152/jn.1998.80.3.1021 9744918

[B139] Rose J. Jin S. X. Craig A. M. (2009). Heterosynaptic molecular dynamics: locally induced propagating synaptic accumulation of CaM kinase II. Neuron 61, 351–358. 10.1016/j.neuron.2008.12.030 19217373 PMC2677100

[B140] Royer S. Paré D. (2003). Conservation of total synaptic weight through balanced synaptic depression and potentiation. Nature 422, 518–522. 10.1038/nature01530 12673250

[B700] Ruthazer E. S. Gillespie D. C. Dawson T. M. Snyder S. H. Stryker M. P. (1996). Inhibition of nitric oxide synthase does not prevent ocular dominance plasticity in kitten visual cortex. J. Physiol. 494, 519–527. 10.10.1113/jphysiol.1996.sp021510 8842009 PMC1160652

[B141] Sahin M. Sur M. (2015). Genes, circuits and precision therapies for autism and related neurodevelopmental disorders. Science 350, 926–934. 10.1126/science.aab3897 26472761 PMC4739545

[B142] Saiepour M. H. Min R. Kamphuis W. Heimel J. A. Levelt C. N. (2018). β-Catenin in the adult visual cortex regulates NMDA-receptor function and visual responses. Cereb. Cortex 28, 1183–1194. 10.1093/cercor/bhx029 28184425

[B143] Sawtell N. B. Frenkel M. Y. Philpot B. D. Nakazawa K. Tonegawa S. Bear M. F. (2003). NMDA receptor-dependent ocular dominance plasticity in adult visual cortex. Neuron 38, 977–985. 10.1016/s0896-6273(03)00323-4 12818182

[B144] Schiller J. Major G. Koester H. J. Schiller Y. (2000). NMDA spikes in basal dendrites. Nature 404, 285–289. 10.1038/35005094 10749211

[B145] Scholl B. Thomas C. I. Ryan M. A. Kamasawa N. Fitzpatrick D. (2021). Cortical response selectivity derives from strength in numbers of synapses. Nature 590, 111–114. 10.1038/s41586-020-03044-3 33328635 PMC7872059

[B146] Scholl B. Wilson D. E. Fitzpatrick D. (2017). Local order within global disorder: synaptic architecture of visual space. Neuron 96, 1127–1138.e4. 10.1016/j.neuron.2017.10.017 29103806 PMC5868972

[B147] Schuman E. M. Madison D. V. Science S. Series N. Jan N. (1994). Locally distributed synaptic potentiation in the hippocampus. Science 263, 532–536. 10.1126/science.8290963 8290963

[B300] Servier Medical Art. (2015). Intracellular Components. Available online at: smart.servier.com. Accessed June 14, 2015.

[B148] Shepherd J. D. Rumbaugh G. Wu J. Chowdhury S. Plath N. Kuhl D.. (2006). Arc/Arg3.1 mediates homeostatic synaptic scaling of AMPA receptors. Neuron 52, 475–484. 10.1016/j.neuron.2006.08.034 17088213 PMC1764219

[B149] Sidorov M. S. Kaplan E. S. Osterweil E. K. Lindemann L. Bear M. F. (2015). Metabotropic glutamate receptor signaling is required for NMDA receptor-dependent ocular dominance plasticity and LTD in visual cortex. Proc. Natl. Acad. Sci. U S A 112, 12852–12857. 10.1073/pnas.1512878112 26417096 PMC4611661

[B150] Singh S. K. Stogsdill J. A. Pulimood N. S. Dingsdale H. Kim Y. H. Pilaz L. J.. (2016). Astrocytes assemble thalamocortical synapses by bridging NRX1α and NL1 *via* hevin. Cell 164, 183–196. 10.1016/j.cell.2015.11.034 26771491 PMC4715262

[B151] Sipe G. O. Petravicz J. Rikhye R. V. Garcia R. Mellios N. Sur M. (2021). Astrocyte glutamate uptake coordinates experience-dependent, eye-specific refinement in developing visual cortex. Glia 69, 1723–1735. 10.1002/glia.23987 33675674 PMC8415121

[B152] Smith S. L. Smith I. T. Branco T. Häusser M. (2013). Dendritic spikes enhance stimulus selectivity in cortical neurons *in vivo*. Nature 503, 115–120. 10.1038/nature12600 24162850 PMC6319606

[B153] Spolidoro M. Putignano E. Munaf C. Maffei L. Pizzorusso T. (2012). Inhibition of matrix metalloproteinases prevents the potentiation of nondeprived-eye responses after monocular deprivation in juvenile rats. Cereb. Cortex 22, 725–734. 10.1093/cercor/bhr158 21685398

[B154] Stellwagen D. Malenka R. C. (2006). Synaptic scaling mediated by glial TNF-α. Nature 440, 1054–1059. 10.1038/nature04671 16547515

[B155] Steward O. Wallace C. S. Lyford G. L. Worley P. F. (1998). Synaptic activation causes the mRNA for the IEG Arc to localize selectively near activated postsynaptic sites on dendrites. Neuron 21, 741–751. 10.1016/s0896-6273(00)80591-7 9808461

[B156] Sun Y. J. Espinosa J. S. Hoseini M. S. Stryker M. P. (2019). Experience-dependent structural plasticity at pre- and postsynaptic sites of layer 2/3 cells in developing visual cortex. Proc. Natl. Acad. Sci. U S A 116, 21812–21820. 10.1073/pnas.1914661116 31591211 PMC6815154

[B158] Taha S. Stryker M. P. (2002). Rapid ocular dominance plasticity requires cortical but not geniculate protein synthesis. Neuron 34, 425–436. 10.1016/s0896-6273(02)00673-6 11988173

[B157] Taha S. A. Stryker M. P. (2005). Ocular dominance plasticity is stably maintained in the absence of α calcium calmodulin kinase II (αCaMKII) autophosphorylation. Proc. Natl. Acad. Sci. U S A 102, 16438–16442. 10.1073/pnas.0508185102 16260732 PMC1283462

[B159] Takahashi N. Kitamura K. Matsuo N. Mayford M. Kano M. Matsuki N.. (2012). Locally synchronized synaptic inputs. Science 335, 353–356. 10.1126/science.1210362 22267814

[B160] Tazerart S. Mitchell D. E. Miranda-Rottmann S. Araya R. (2020). A spike-timing-dependent plasticity rule for dendritic spines. Nat. Commun. 11:4276. 10.1038/s41467-020-17861-7 32848151 PMC7449969

[B161] Tong R. Chater T. E. Emptage N. J. Goda Y. (2021). Heterosynaptic cross-talk of pre- and postsynaptic strengths along segments of dendrites. Cell Rep. 34:108693. 10.1016/j.celrep.2021.108693 33503435

[B162] Trachtenberg J. T. Chen B. E. Knott G. W. Feng G. Sanes J. R. Welker E.. (2002). Long-term *in vivo* imaging of experience-dependent synaptic plasticity in adult cortex. Nature 420, 788–794. 10.1038/nature01273 12490942

[B163] Tropea D. Giacometti E. Wilson N. R. Beard C. McCurry C. Dong D. F.. (2009a). Partial reversal of Rett Syndrome-like symptoms in MeCP2 mutant mice. Proc. Natl. Acad. Sci. U S A 106, 2029–2034. 10.1073/pnas.0812394106 19208815 PMC2644158

[B165] Tropea D. Van Wart A. Sur M. (2009b). Molecular mechanisms of experience-dependent plasticity in visual cortex. Philos. Trans. R. Soc. B. Biol. Sci. 364, 341–355. 10.1098/rstb.2008.0269 18977729 PMC2674480

[B164] Tropea D. Kreiman G. Lyckman A. Mukherjee S. Yu H. Horng S.. (2006). Gene expression changes and molecular pathways mediating activity-dependent plasticity in visual cortex. Nat. Neurosci. 9, 660–668. 10.1038/nn1689 16633343

[B166] Tropea D. Majewska A. K. Garcia R. Sur M. (2010). Structural dynamics of synapses *in vivo* correlate with functional changes during experience-dependent plasticity in visual cortex. J. Neurosci. 30, 11086–11095. 10.1523/JNEUROSCI.1661-10.2010 20720116 PMC2932955

[B167] Tsukamoto M. Yasui T. Yamada M. K. Nishiyama N. Matsuki N. Ikegaya Y. (2003). Mossy fibre synaptic NMDA receptors trigger non-Hebbian long-term potentiation at entorhino-CA3 synapses in the rat. J. Physiol. 546, 665–675. 10.1113/jphysiol.2002.033803 12562995 PMC2342574

[B168] Turrigiano G. G. Nelson S. B. (2004). Homeostatic plasticity in the developing nervous system. Nat. Rev. Neurosci. 5, 97–107. 10.1038/nrn1327 14735113

[B169] Uebele V. N. Nuss C. E. Santarelli V. P. Garson S. L. Barrow J. C. Stauffer S. R.. (2009). T-type calcium channels regulate cortical plasticity *in-vivo*. Neuroreport 20, 257–262. 10.1097/WNR.0b013e3283200111 19212242 PMC2902375

[B171] van Versendaal D. Rajendran R. Saiepour H. M. Klooster J. Smit-Rigter L. Sommeijer J. P.. (2012). Elimination of inhibitory synapses is a major component of adult ocular dominance plasticity. Neuron 74, 374–383. 10.1016/j.neuron.2012.03.015 22542189

[B173] Wang B.-S. Feng L. Liu M. Liu X. Cang J. (2013). Environmental enrichment rescues binocular matching of orientation preference in mice that have a precocious critical period. Neuron 80, 198–209. 10.1016/j.neuron.2013.07.023 24012279 PMC3796011

[B200] Wang B.-S. Sarnaik R. Cang J. (2010). Critical period plasticity matches binocular orientation preference in the visual cortex. Neuron 65, 246–256. 10.1016/j.neuron.2010.01.002 20152130 PMC2822731

[B174] Waung M. W. Pfeiffer B. E. Nosyreva E. D. Ronesi J. A. Huber K. M. (2008). Rapid translation of Arc/Arg3.1 selectively mediates mGluR-dependent LTD through persistent increases in AMPAR endocytosis rate. Neuron 59, 84–97. 10.1016/j.neuron.2008.05.014 18614031 PMC2580055

[B175] Weber J. P. Andrásfalvy B. K. Polito M. Magó Á. Ujfalussy B. B. Makara J. K. (2016). Location-dependent synaptic plasticity rules by dendritic spine cooperativity. Nat. Commun. 7:11380. 10.1038/ncomms11380 27098773 PMC4844677

[B176] White G. Levy W. B. Steward O. (1990). Spatial overlap between populations of synapses determines the extent of their associative interaction during the induction of long-term potentiation and depression. J. Neurophysiol. 64, 1186–1198. 10.1152/jn.1990.64.4.1186 2258741

[B177] Wilkerson J. R. Tsai N.-P. Maksimova M. A. Wu H. Cabalo N. P. Loerwald K. W.. (2014). A role for dendritic mGluR5-mediated local translation of Arc/Arg3.1 in MEF2-dependent synapse elimination. Cell Rep. 7, 1589–1600. 10.1016/j.celrep.2014.04.035 24857654 PMC4057996

[B178] Wilson D. E. Whitney D. E. Scholl B. Fitzpatrick D. (2016). Orientation selectivity and the functional clustering of synaptic inputs in primary visual cortex. Nat. Neurosci. 19, 1003–1009. 10.1038/nn.4323 27294510 PMC5240628

[B179] Winnubst J. Cheyne J. E. Niculescu D. Lohmann C. (2015). Spontaneous activity drives local synaptic plasticity *in vivo*. Neuron 87, 399–410. 10.1016/j.neuron.2015.06.029 26182421

[B180] Xu T. Yu X. Perlik A. J. Tobin W. F. Zweig J. A. Tennant K.. (2009). Rapid formation and selective stabilization of synapses for enduring motor memories. Nature 462, 915–919. 10.1038/nature08389 19946267 PMC2844762

[B182] Yang Y. Fischer Q. S. Zhang Y. Baumgärtel K. Mansuy I. M. Daw N. W. (2005). Reversible blockade of experience-dependent plasticity by calcineurin in mouse visual cortex. Nat. Neurosci. 8, 791–796. 10.1038/nn1464 15880107

[B181] Yang G. Lai C. S. W. Cichon J. Ma L. Li W. Gan W. B. (2014). Sleep promotes branch-specific formation of dendritic spines after learning. Science 344, 1173–1178. 10.1126/science.1249098 24904169 PMC4447313

[B183] Yang Y. Liu D. Huang W. Deng J. Sun Y. Zuo Y.. (2016). Selective synaptic remodeling of amygdalocortical connections associated with fear memory. Nat. Neurosci. 19, 1348–1355. 10.1038/nn.4370 27595384

[B184] Yoon B.-J. Smith G. B. Heynen A. J. Neve R. L. Bear M. F. (2009). Essential role for a long-term depression mechanism in ocular dominance plasticity. Proc. Natl. Acad. Sci. U S A 106, 9860–9865. 10.1073/pnas.0901305106 19470483 PMC2685742

[B185] Yoshimura Y. Inaba M. Yamada K. Kurotani T. Begum T. Reza F.. (2008). Involvement of T-type Ca2+ channels in the potentiation of synaptic and visual responses during the critical period in rat visual cortex. Eur. J. Neurosci. 28, 730–743. 10.1111/j.1460-9568.2008.06384.x 18657180

[B186] Zuo Y. Lin A. Chang P. Gan W. B. (2005a). Development of long-term dendritic spine stability in diverse regions of cerebral cortex. Neuron 46, 181–189. 10.1016/j.neuron.2005.04.001 15848798

[B187] Zuo Y. Yang G. Kwon E. Gan W. B. (2005b). Long-term sensory deprivation prevents dendritic spine loss in primary somatosensory cortex. Nature 436, 261–265. 10.1038/nature03715 16015331

